# Apolipoprotein E promotes subretinal mononuclear phagocyte survival and chronic inflammation in age-related macular degeneration

**DOI:** 10.15252/emmm.201404524

**Published:** 2015-01-20

**Authors:** Olivier Levy, Bertrand Calippe, Sophie Lavalette, Shulong J Hu, William Raoul, Elisa Dominguez, Michael Housset, Michel Paques, José-Alain Sahel, Alexis-Pierre Bemelmans, Christophe Combadiere, Xavier Guillonneau, Florian Sennlaub

**Affiliations:** 1 INSERMParis, France; 2 UPMC Univ Paris 06, UMR_S 968, Institut de la VisionParis, France; 3 Centre Hospitalier National d'Ophtalmologie des Quinze-Vingts, INSERM-DHOS CIC 503Paris, France; 4 CEA, DSV, I²BM, Molecular Imaging Research Center (MIRCen)Fontenay-aux-Roses, France; 5 CNRS, CEA URA 2210Fontenay-aux-Roses, France; 6 Sorbonne Universités, UPMC Univ Paris 06, CR7, Centre d'Immunologie et des Maladies Infectieuses (CIMI-Paris)Paris, France; 7 INSERM, U1135, CIMI-ParisParis, France; 8 CNRS, ERL 8255, CIMI-ParisParis, France

**Keywords:** age-related macular degeneration, apolipoprotein E, interleukin 6, mononuclear phagocyte, neuroinflammation

## Abstract

Physiologically, the retinal pigment epithelium (RPE) expresses immunosuppressive signals such as FAS ligand (FASL), which prevents the accumulation of leukocytes in the subretinal space. Age-related macular degeneration (AMD) is associated with a breakdown of the subretinal immunosuppressive environment and chronic accumulation of mononuclear phagocytes (MPs). We show that subretinal MPs in AMD patients accumulate on the RPE and express high levels of APOE. MPs of *Cx3cr1*^−/−^ mice that develop MP accumulation on the RPE, photoreceptor degeneration, and increased choroidal neovascularization similarly express high levels of APOE. *ApoE* deletion in *Cx3cr1*^−/−^ mice prevents pathogenic age- and stress-induced subretinal MP accumulation. We demonstrate that increased APOE levels induce IL-6 in MPs via the activation of the TLR2-CD14-dependent innate immunity receptor cluster. IL-6 in turn represses RPE FasL expression and prolongs subretinal MP survival. This mechanism may account, in part, for the MP accumulation observed in *Cx3cr1*^−/−^ mice. Our results underline the inflammatory role of APOE in sterile inflammation in the immunosuppressive subretinal space. They provide rationale for the implication of IL-6 in AMD and open avenues toward therapies inhibiting pathogenic chronic inflammation in late AMD.

## Introduction

Age-related macular degeneration (AMD) is the leading cause of irreversible blindness in the industrialized world (Klein *et al*, 2004). Early and intermediate AMD is characterized by sizeable (> 125 μm) deposits of lipoproteinaceous debris, called large drusen, which are located in the Bruch's membrane (BM) and are partially covered by the retinal pigment epithelium (RPE) (Sarks, 1976). The presence of large drusen is an important risk factor for late AMD (Klein *et al*, 2004). There are two clinical forms of late AMD: wet AMD, which is defined by choroidal neovascularization (CNV), and geographic atrophy (GA), which is characterized by an extending lesion of both the retinal pigment epithelium (RPE) and photoreceptors (Sarks, 1976).

Mononuclear phagocytes (MP) comprise a family of cells that include microglial cells (MC), monocytes (Mo), and macrophages (Mϕ) among others (Chow *et al*, 2011). Physiologically, MCs are present only in the inner retina. A number of neuron- and glial-derived factors (e.g., CX3CL1, CD200L) repress their activation (Galea *et al*, 2007). The subretinal space, located between the RPE and the photoreceptor outer segments (POS), is a zone of immune privilege mediated by immunosuppressive RPE signals, including leukocyte-suppressing FasL (CD95L) (Griffith *et al*, 1995; Streilein *et al*, 2002). In healthy subjects, the subretinal space is physiologically devoid of all leukocytes, including MPs, but in both advanced forms of AMD, wet AMD and geographic atrophy (GA), MPs accumulate in the subretinal space (Oh *et al*, 1999; Gupta *et al*, 2003; Combadiere *et al*, 2007; Sennlaub *et al*, 2013). They are in close contact with the RPE in CNV and in the vicinity of the RPE lesion in GA (Oh *et al*, 1999; Gupta *et al*, 2003; Combadiere *et al*, 2007; Sennlaub *et al*, 2013). It is generally admitted that MPs play an important role in CNV (Sakurai *et al*, 2003; Tsutsumi *et al*, 2003). Recent evidence strongly suggests that CCL2/CCR2-dependent subretinal MP accumulation is an important contributing factor in photoreceptor degeneration in models of photo-oxidative stress (Rutar *et al*, 2012; Suzuki *et al*, 2012), in the *Abca4*^−/−^*Rdh8*^−/−^ mouse Stargardt/AMD model (Kohno *et al*, 2013), in the carboxyethylpyrrole immunization-induced AMD model (Cruz-Guilloty *et al*, 2013), and in the age- and light-induced photoreceptor degeneration of *Cx3cr1*^−/−^ mice (Combadiere *et al*, 2007; Sennlaub *et al*, 2013). The reasons for the breakdown of subretinal immunosuppression and accumulation of MPs in AMD remain unknown.

Apolipoprotein E (APOE) plays a crucial role in lipid transport (Mahley & Rall, 2000). APOE is expressed in the liver (Linton *et al*, 1995), is a major component of high-density lipoproteins (HDL) of the blood (Mahley & Rall, 2000), and is the main lipoprotein of the brain and the retina (Mahley & Rall, 2000; Anderson *et al*, 2001). APOE is also strongly expressed in MCs (Nakai *et al*, 1996; Peri & Nusslein-Volhard, 2008) and Mϕs (Basu *et al*, 1982; Rosenfeld *et al*, 1993) and promotes macrophage lipid efflux (Matsuura *et al*, 2006). In conjunction with APOA-I, APOE plays a crucial role in reverse cholesterol transport (Mahley & Rall, 2000). It also binds lipopolysaccharide (LPS) (Berbee *et al*, 2005) and amyloid-β (Zhao *et al*, 2009) and inhibits their activation of Toll-like receptors (TLR) and the induction of inflammatory mediators similar to APOA-I (Guo *et al*, 2004; Ali *et al*, 2005). TLR2 and TLR4 signaling necessitates their recruitment to the CD14-dependent innate immunity receptor cluster, located in cholesterol-rich membrane domains called lipid rafts (Schmitz & Orso, 2002). Interestingly, APOA-I and APOE can also extract membrane cholesterol from lipid rafts, activate the innate immunity receptor cluster in the absence of TLR ligands, and induce several inflammatory cytokines including IL-6 (shown for APOA-I) (Smoak *et al*, 2010). Recently, it has been shown that increased IL-6 levels are associated with AMD incidence (Klein *et al*, 2014) and with late AMD (Seddon *et al*, 2005; Klein *et al*, 2008). It is currently not clear how APOE and IL-6 participate in AMD pathogenesis.

In humans, *APOE* exists in three isoforms (*APOE2*, *APOE3*, and *APOE4*) arising from two cysteine–arginine interchanges at residues 112 and 158. Homozygous *APOE2*-allele carriers are at increased risk for developing late AMD (recently confirmed in 20,000 subjects (McKay *et al*, 2011)) and are protected against Alzheimer's disease (AD), while the *APOE4* allele protects against AMD and is a risk factor for AD (Mahley & Rall, 2000).

The risk-conferring property of the *APOE2* allele might be due to a difference in quantity or result from functional differences of the APOE2 protein structure. APOE concentrations in plasma and cerebro-spinal fluid (CSF) of *APOE2* humans and transgenic humanized mice are significantly higher than in *APOE3* carriers (Mooijaart *et al*, 2006; Riddell *et al*, 2008). This difference is due to APOE2's decreased ability to bind and be cleared via the LDL receptor (Mahley & Rall, 2000). Consistently increased APOE immunostaining in eyes with AMD would also suggest that APOE concentrations are locally increased in AMD (Klaver *et al*, 1998; Anderson *et al*, 2001).

On the other hand, *ApoE*^−/−^ mice that are fed a high-fat diet develop lipid accumulations in BM, which are proposed as being similar to early AMD (Ong *et al*, 2001). *APOE4* transgenic mice on high-fat diet develop similar deposits (Malek *et al*, 2005) even though the APOE concentration observed in *APOE4* transgenic mice is only marginally decreased in their plasma (Riddell *et al*, 2008) and similar in the CSF (Riddell *et al*, 2008) and retina (Malek *et al*, 2005) compared to *APOE3* mice. The structural changes in the APOE4 protein, however, lead to diminished association with HDL (Dong & Weisgraber, 1996) and impaired reverse cholesterol transport (Heeren *et al*, 2004). Low APOE concentrations or impaired reverse cholesterol transport could thereby hinder efficient lipid evacuation from the RPE to the choroid and lead to drusen development. (Mahley & Rall, 2000; Mooijaart *et al*, 2006; Riddell *et al*, 2008; Malek *et al*, 2005). This hypothesis is, however, in contradiction with the APOE accumulation observed in AMD donor eyes (Klaver *et al*, 1998; Anderson *et al*, 2001) and the protective effect of the *APOE4* allele in AMD (McKay *et al*, 2011).

In the present article, we show that subretinal MPs in AMD and in subretinal inflammation observed in *Cx3cr1*^−/−^ mice strongly express APOE. We demonstrate that APOE prolongs subretinal MP survival and is necessary for subretinal MP accumulation in *Cx3cr1*^−/−^ mice. We demonstrate that increased APOE induces IL-6 in MPs, via the activation of the TLR2-CD14-dependent innate immune receptor cluster. IL-6 in turn represses RPE FasL expression, prolongs subretinal MP survival, and promotes chronic subretinal inflammation. Our data suggest that CD14 or IL-6 inhibition can help reestablish RPE immune-suppressive function and inhibit pathogenic inflammation in late AMD.

## Results

### Subretinal MPs accumulate on the RPE in the vicinity of atrophic lesions and large drusen

**Figure 1:**
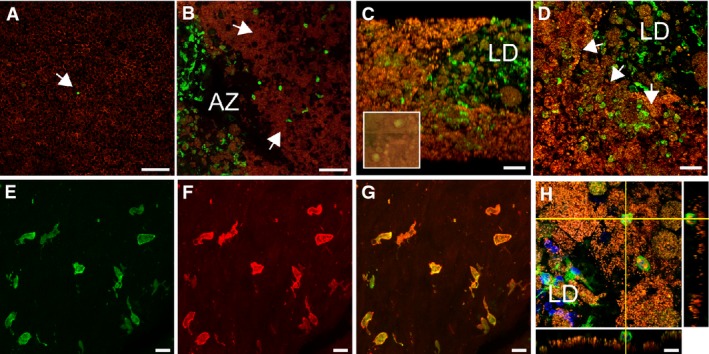
Subretinal MPs accumulate on the retinal pigment epithelium in AMD

Physiologically, the subretinal space does not contain significant numbers of MPs, possibly due in part to immunosuppressive RPE signals (Streilein *et al*, 2002). In late AMD, immunohistochemical studies on sections revealed the presence of subretinal MPs on RPE cells adjacent to the lesions of atrophic AMD (Gupta *et al*, 2003; Sennlaub *et al*, 2013) and MPs are found in subretinal neovascular membranes (Oh *et al*, 1999). Because the small, dispersed MPs are difficult to detect on sections, we performed MP marker IBA-1 immunohistochemistry on healthy and diseased macular RPE/choroidal flatmounts (IBA-1 green fluorescence, RPE autofluorescence visible as orange due to its autofluorescence in the red and green channel). Confocal microscopy confirmed that subretinal IBA-1^+^ MPs are only very occasionally observed in healthy age-matched donor central RPE (Fig1A). Within the atrophic lesions of GA patients where the RPE has disappeared, MPs were numerous, but were also invariably observed on the apical side of the RPE adjacent to the lesions (Fig1B). Furthermore, IBA-1^+^ cells were detected on the RPE adjacent to large drusen (> 125 μm), visible under the dissecting microscope as pale lesions after removal of the retina (Fig1C, inset) and as dome-shaped protrusions under the confocal microscope (Fig1C, oblique projection of a Z-stack; Fig1D, orthogonal Z-stack projection). Double-labeling on the subretinal side of the overlaying retina (to avoid masking by RPE autofluorescence) shows that subretinal IBA-1^+^ MPs (Fig1E green fluorescence) also express the pan-MP marker CD18 (Fig1F red fluorescence, Fig1G merge). IBA-1^+^ MPs in close contact with the RPE (Fig1H, lateral Z-stack projections) were observed in the vicinity of all examined large drusen and atrophic zones. Interestingly, we also detected IBA-1^+^ MPs within the large drusen, confirming our previous immunohistochemical detection of CCR2^+^ MPs on paraffin sections within large drusen (Sennlaub *et al*, 2013). HLA-DR and CD68-positive MP dendrites have previously been observed in smaller, dome-shaped drusen (Hageman *et al*, 2001). A detailed analysis of MPs within drusen of different sizes is ongoing in our laboratory, but beyond the scope of this study.

Confocal microscopy of IBA-1 (green staining) immunohistochemistry of RPE flatmounts (RPE autofluorescence visible as orange due to its autofluorescence in the red and green channel) from a healthy donor (A), a geographic atrophy lesion (B), and large drusen (C and D). (A, B, D): orthogonal *Z*-stack projection; (C): oblique Z-stack projection and dissecting microscope appearance of postmortem large drusen after removal of the overlaying retina (inset).

Double-labeling on the subretinal side of the retina (to avoid masking by RPE autofluorescence) of IBA-1^+^ (E, green fluorescence) and CD18 (F, red fluorescence; G, merge).

Orthogonal and lateral Z-stack of a subretinal IBA-1^+^ (green fluorescence) MPs adjacent to the RPE (orange autofluorescence) in the vicinity of a large drusen. Data information: Controls omitting the primary antibody showed no staining apart from the autofluorescence. AZ: atrophic zone; LD: large drusen. Scale bar: 50 μm (A–D); 10 μm (E–H).

These observations considered together confirm the presence of subretinal MPs in AMD (Penfold *et al*, 1985; Gupta *et al*, 2003; Sennlaub *et al*, 2013) and illustrate their presence in contact with the RPE around large drusen and GA lesions. They are very rare in healthy donors. This further suggests that RPE-mediated immunosuppression is impaired in intermediate AMD (large drusen) and late AMD (GA).

### Subretinal MPs express APOE

**Figure 2:**
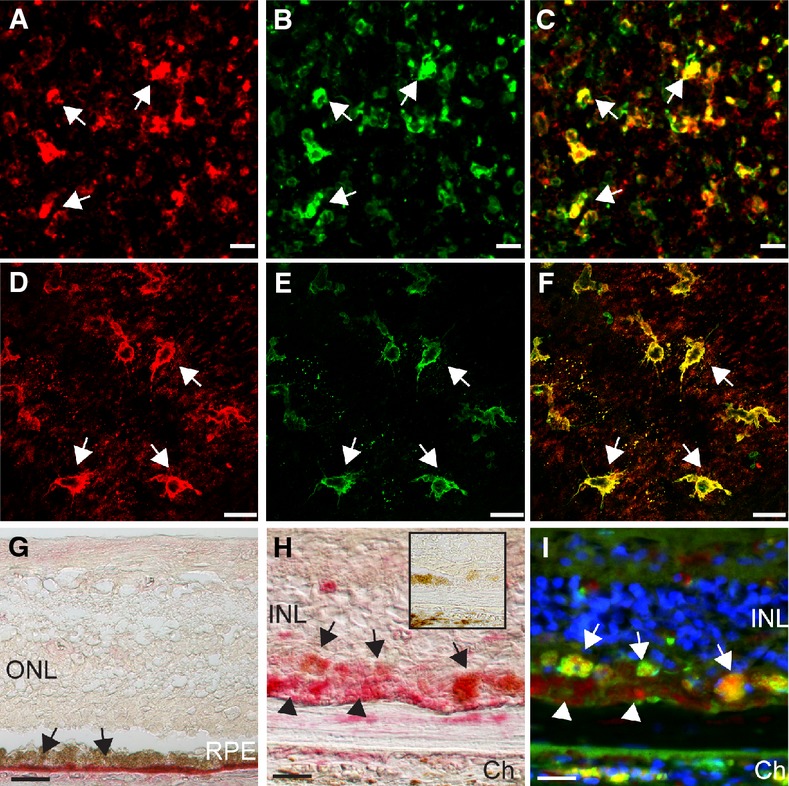
Subretinal MPs express APOE

MPs have been reported to express APOE at high levels (Basu *et al*, 1982; Rosenfeld *et al*, 1993; Nakai *et al*, 1996; Peri & Nusslein-Volhard, 2008). Immunohistochemistry of APOE (Fig2A, red) and IBA-1 (Fig2B, green; Fig 2C, merge) on paraffin sections of human tonsils, which we used as a positive control, confirmed that IBA-1^+^ MPs can strongly express APOE. Similarly, on retinal flatmounts of donor eyes with large drusen, APOE (Fig2D, red) staining was observed in and around subretinal IBA-1^+^ MPs (Fig2E, green; Fig 2F, merge). The double-labeling was performed on the subretinal side of retinas to avoid masking by the RPE autofluorescence. We next performed APOE staining on paraffin sections of controls and donor eyes with geographic atrophy lesions. We used a substrate revealing method (alkaline phosphatase/Fast Red) that is visible in bright field to circumvent confusion with RPE autofluorescence. In sections from control eyes, the APOE signal was concentrated at the basal portion of the RPE (Fig2G red signal, arrows). In donor eyes with GA, adjacent to the atrophic area, a strong APOE signal was observed in the RPE, but it was less restricted to the basal aspect than in controls (Fig2H APOE red signal, arrowheads). Additionally, APOE immunostaining was observed in cells adjacent to the RPE (Fig2H APOE red signal, arrows). Double-labeling with IBA-1 identified these cells as subretinal IBA-1^+^ MPs (Fig2I IBA-1 green signal, arrows). Omitting the APOE antibody and following the same experimental protocol did not produce any significant staining (Fig2H inset).

APOE (A) and IBA-1 (B, merge in C) double-labeling on paraffin sections of surgical specimens of human tonsils.

APOE (D) and IBA-1 (E, merge in F) double-labeling on the subretinal aspect of a large drusen overlaying retina (to avoid specific signal masking by the RPE orange autofluorescence).

APOE (red staining) immunohistochemistry of control donor tissue (G) and adjacent to GA lesion (H).

IBA-1 (green staining, RPE far-red autofluorescence) double-labeling of the GA lesion shown in (H). Representative images from five donor eyes with large drusen, five donor eyes with GA, and three healthy donors between 70 and 89 years old. Data information: Controls omitting the primary antibody showed no staining apart from the autofluorescence (H inset). ONL: outer nuclear layer; INL: inner nuclear layer; Ch: choroid. Scale bar: 10 μm.

Taken together, our results show that, in addition to the RPE, subretinal MPs in AMD patients strongly express APOE in a manner similar to other inflammatory settings (e.g., atherosclerotic lesions (Rosenfeld *et al*, 1993)).

### APOE promotes subretinal MP accumulation in *Cx3cr1^GFP/GFP^* mice

In the eye, CX3CL1 is constitutively expressed as a transmembrane protein in inner retinal neurons (Silverman *et al*, 2003; Zieger *et al*, 2014) and provides a tonic inhibitory signal to CX3CR1 bearing retinal MCs that keeps these cells in a quiescent surveillance mode under physiological conditions (Combadiere *et al*, 2007; Ransohoff, 2009). *Cx3cr1* deficiency in mice leads to a strong increase of subretinal MP accumulation with age, after light challenge or laser injury (Combadiere *et al*, 2007; Raoul *et al*, 2008; Ma *et al*, 2009), in diabetes (Kezic *et al*, 2013) and in a paraquat-induced retinopathy model (Chen *et al*, 2013). *Cx3cr1*^*GFP/GFP*^ mice do not develop drusen and RPE atrophy, but do model MP accumulation on the RPE, as well as the associated photoreceptor degeneration and excessive CNV observed in AMD (Combadiere *et al*, 2007; Sennlaub *et al*, 2013).

**Figure 3:**
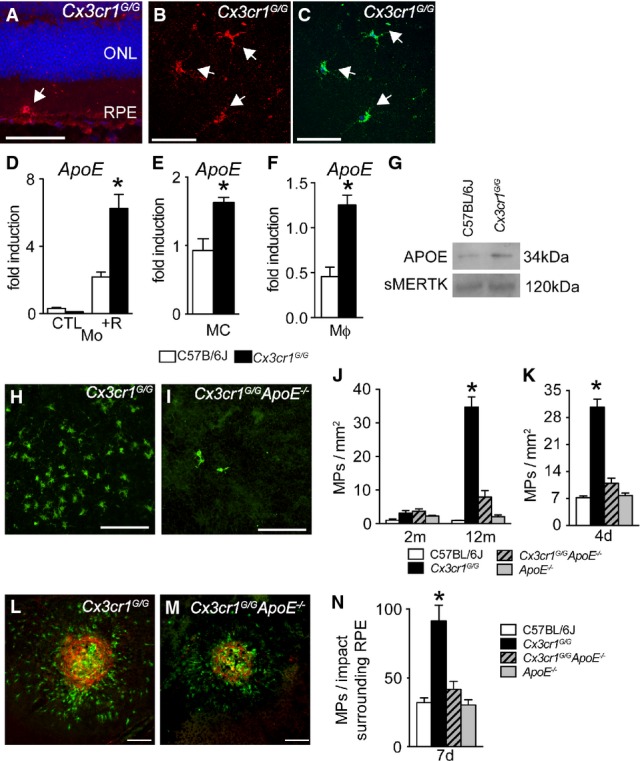
APOE promotes subretinal MP accumulation in *Cx3cr1^GFP/GFP^* mice

We first evaluated APOE localization in 12-month-old *Cx3cr1*^*GFP/GFP*^ mice that present subretinal MP accumulation (Sennlaub *et al*, 2013). Immunohistochemical localization of APOE on retinal sections of both 12-month-old wild-type and *Cx3cr1*^*GFP/GFP*^ mice revealed APOE localization mainly in the RPE and inner retina as previously described (Anderson *et al*, 2001) (Supplementary Fig S1). Additionally, we detected a strong signal in cells apposed to the RPE on retinal sections and the subretinal side of retinal flatmounts in aged *Cx3cr1*^*GFP/GFP*^ mice (arrow, Fig3A and B, red) that were identified as IBA-1-expressing MPs (Fig3C, green), similar to AMD patients (Fig1).

Immunohistochemistry of APOE (red) and IBA-1 (green) on a section (A, blue Hoechst) and the subretinal side of a retinal flatmount (B and C) from a 12-month-old *Cx3cr1*^*GFP*^^*/*^^*GFP*^ mouse (representative of 3 independent experiments, experiments omitting the primary antibody immunostaining served as negative controls). ONL: outer nuclear layer; RPE: retinal pigment epithelium.

Quantitative RT–PCR of *ApoE*mRNA normalized with *S26*mRNA of C57BL/6J and *Cx3cr1*^*GFP*^^*/*^^*GFP*^ monocytes cultured for 24 h in contact with POS of an overlaying retinal explant (*n* = 4/group per experiment; Mann–Whitney *U*-test, *P = 0.0286, the experiment was repeated twice with similar results).

Quantitative RT–PCR of *ApoE*mRNA normalized with *S26*mRNA of C57BL/6J and *Cx3cr1*^*GFP*^^*/*^^*GFP*^ FACS-sorted microglial cells, freshly extracted from adult brain (*n* = 4–5/group; Mann–Whitney *U*-test, C57BL/6J versus *Cx3cr1^GFP/GFP^* *P = 0.0159).

Quantitative RT–PCR of *ApoE*mRNA normalized with *S26*mRNA of C57BL/6J and *Cx3cr1*^*GFP*^^*/*^^*GFP*^ peritoneal macrophages cultured for 24 h with CX3CL1. (*n* = 4/group per experiment; Mann–Whitney *U*-test, *P = 0.0286. The experiment was repeated twice with similar results).

APOE Western blot analysis of equivalent amounts of supernatant protein from CX3CL1-exposed C57BL/6J and *Cx3cr1*^*GFP*^^*/*^^*GFP*^ peritoneal macrophages at 24 h. Soluble Mer receptor tyrosine kinase that is released constitutively from cultured macrophages served as a loading control. The experiment was repeated twice with similar results.

12-month-old IBA-1 stained RPE flatmounts of *Cx3cr1*^*GFP*^^*/*^^*GFP*^ (H) and *Cx3cr1*^*GFP*^^*/*^^*GFP*^*ApoE*^*−/−*^ (I) mice.

Quantification of subretinal IBA-1^+^ mononuclear phagocytes in 2-month-old (left) and 12-month-old (right) mice of the indicated strains (*n* = 9–20/group ANOVA/Dunnett test at 12 months: *Cx3cr1*^*GFP*^^*/*^^*GFP*^ versus any other group *P < 0.0001; Mann–Whitney *U*-test at 12 months of *Cx3cr1*^*GFP*^^*/*^^*GFP*^ versus *Cx3cr1*^*GFP*^^*/*^^*GFP*^*ApoE*^−/−^ *P < 0.0001).

Quantification of subretinal IBA-1^+^ mononuclear phagocytes after 4 days of light challenge of 2-month-old mice of the indicated strains (*n* = 10–25/group ANOVA/Dunnett test: *Cx3cr1*^*GFP*^^*/*^^*GFP*^ versus any other group *P < 0.0001; Mann–Whitney *U*-test of *Cx3cr1*^*GFP*^^*/*^^*GFP*^ versus *Cx3cr1*^*GFP*^^*/*^^*GFP*^*ApoE*^−/−^ *P < 0.0001).

IBA-1- (green) and CD102- (red) stained RPE flatmounts 7 days after laser injury of 2-month-old *Cx3cr1*^*GFP*^^*/*^^*GFP*^ (E) and *Cx3cr1*^*GFP*^^*/*^^*GFP*^*ApoE*^−/−^ (F) mice.

Quantification of subretinal IBA-1^+^ mononuclear phagocytes on the RPE counted on the RPE at a distance of 0–500 μm to CD102^+^ CNV 7 days after the laser injury of 2-month-old mice of the indicated strains (*n* = 8–10 eyes/group ANOVA/Dunnett test: *Cx3cr1*^*GFP*^^*/*^^*GFP*^ versus any other group *P < 0.0001; Mann–Whitney *U*-test of *Cx3cr1*^*GFP*^^*/*^^*GFP*^ versus *Cx3cr1*^*GFP*^^*/*^^*GFP*^*ApoE*^−/−^ *P < 0.0001). Data information: +R: cultured with an overlying retinal explant. Scale bars, 50 μm.

Subretinal MPs are derived from both Mos and MCs (Sennlaub *et al*, 2013). To evaluate whether *Cx3cr1*^*GFP/GFP*^ MPs differ in their *ApoE* expression, we first studied WT- and *Cx3cr1*^*GFP/GFP*^-Mo (prepared from bone marrow) cultured for 24 h in contact with the photoreceptor outer segment (POS) of an overlaying retinal explant to simulate MP differentiation in the subretinal space. *ApoE* mRNA was expressed at significantly higher levels in *Cx3cr1*^*GFP/GFP*^*-*Mos in the presence of POS of an overlaying retinal explant (Fig3D). Similarly, significantly increased amounts of *ApoE* mRNA were also observed in FACS-sorted MCs freshly extracted from adult *Cx3cr1*^*GFP/GFP*^ brain (Fig3E). The expression of *ApoE* mRNA was also significantly higher in *Cx3cr1*^*GFP/GFP*^ peritoneal Mϕs (prepared from thioglycollate-elicited peritonitis) when compared to WT-Mϕs cultured for 24 h in the presence of CX3CL1 (Fig3F). Western blot analysis of equivalent amounts of supernatant protein from peritoneal Mϕs also showed increased APOE secretion (Fig3G) in the *Cx3cr1*^*GFP/GFP*^ samples when compared to the soluble Mer receptor tyrosine kinase that is released constitutively from cultured macrophages (Sather *et al*, 2007) and which served here as a loading control.

To evaluate the role of APOE in subretinal MP accumulation, we analyzed *Cx3cr1*^*GFP/GFP*^ *ApoE*^−/−^ mice. Quantification of subretinal IBA-1^+^ MPs on retinal and RPE/choroidal flatmounts of 12-month-old *Cx3cr1*^*GFP/GFP*^ (Fig3H) and *Cx3cr1*^*GFP/GFP*^ *ApoE*^−/−^ mice (Fig2I) showed that the significant age-dependent subretinal MP accumulation observed in *Cx3cr1*^*GFP/GFP*^ mice was nearly completely inhibited in *Cx3cr1*^*GFP/GFP*^ *ApoE*^−/−^ mice (Fig3J). Similarly, *Cx3cr1*^*GFP/GFP*^ *ApoE*^−/−^ mice were significantly protected against the subretinal MP accumulation observed in *Cx3cr1*^*GFP/GFP*^ mice after 4 days of light challenge (Fig3K). It should be noted that the intensity of the light challenge model used herein is sufficient to induce subretinal inflammation in the *Cx3cr1*^−/−^ mice but does not cause significant subretinal inflammation nor degeneration in WT mice (Sennlaub *et al*, 2013). Moreover, 7 days after a laser impact, subretinal IBA-1^+^ MPs (green staining) counted on the RPE at a distance of 0–500 μm to CD102^+^ CNV (red staining) in *Cx3cr1*^*GFP/GFP*^ (Fig3L) and *Cx3cr1*^*GFP/GFP*^ *ApoE*^−/−^ mice (Fig3M) were significantly inhibited in *Cx3cr1*^*GFP/GFP*^ *ApoE*^−/−^ mice (Fig2N). Additionally, *ApoE* deletion also significantly inhibited the age-dependent photoreceptor degeneration and exaggerated CNV observed in *Cx3cr1*^*GFP/GFP*^ mice (Supplementary Figs S2 and S3).

C57BL/6 mice are inbred and can carry *Pde6b*^*rd1*^ (retinal degeneration 1), *Crb1*^*rd8*^ (retinal degeneration 8), and *Gnat2*^*cpfl3*^ (Cone photoreceptor function loss 3) mutations relatively commonly (Chang *et al*, 2013). These mutations can lead to subretinal inflammation secondary to primary retinal degeneration (Luhmann *et al*, 2012). In our experiments, all mice strains used tested negative for these three mutations. Furthermore, subretinal MP accumulation in 12-month-old *Cx3cr1*^*+/GFP*^ and *Cx3cr1*^*GFP/GFP*^ littermates of *Cx3cr1*^*+/GFP*^ breeders showed no evidence of influence from an unknown contributor gene specific to the *Cx3cr1*^*GFP/GFP*^ mouse line (Supplementary Fig S4). *Cx3cr1*^*GFP/GFP*^ *ApoE*^−/−^ mice were generated twice with independently purchased *Cx3cr1*^*GFP/GFP*^ and *ApoE*^−/−^ mice (once at the Laboratoire Immunité et Infection and once at the Institut de la Vision), and both *Cx3cr1*^*GFP/GFP*^ *ApoE*^−/−^ mice strain generations were protected against the subretinal MP accumulation observed in the two *Cx3cr1*^*GFP/GFP*^ mouse strains of the two sites. Taken together, these results make it highly unlikely that the MP accumulation in *Cx3cr1*^*GFP/GFP*^ mice and the protection in *Cx3cr1*^*GFP/GFP*^ *ApoE*^−/−^ mice are due to genes other than *Cx3cr1* and *ApoE*.

In summary, we show that APOE is robustly expressed in subretinal MPs, more strongly expressed in *Cx3cr1*^*GFP/GFP*^ MPs, and that *ApoE* deletion very significantly inhibited the age-, light-, and laser-induced accumulation of subretinal MPs observed in *Cx3cr1*-deficient mice.

### APOE inhibits subretinal MP clearance

**Figure 4:**
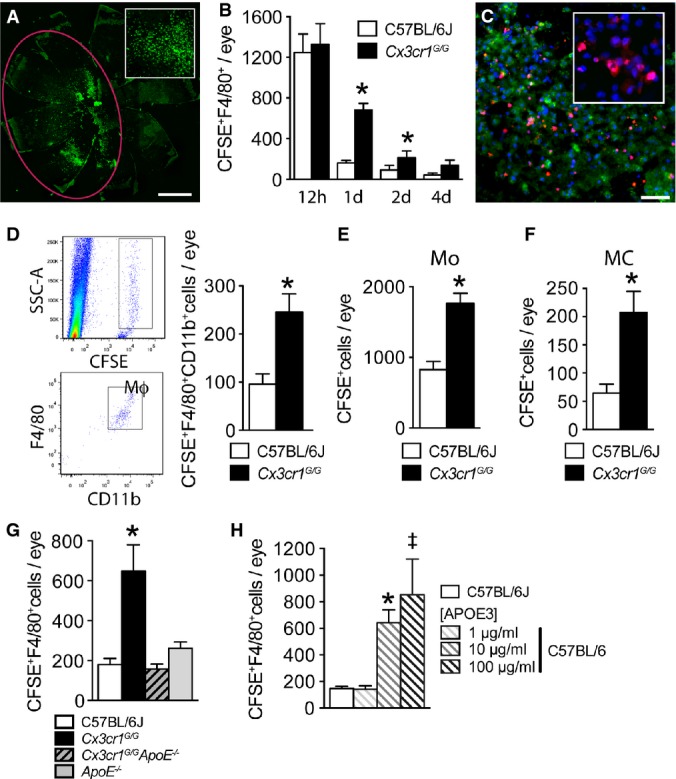
APOE inhibits subretinal MP clearance

The reasons for which subretinal MPs accumulate in *Cx3cr1*-deficient mice are not fully understood. Theoretically, the numbers of subretinal MPs are determined by (i) recruitment, (ii) *in situ* proliferation, (iii) migration (egress), and/or (iv) apoptotic clearance. We previously showed that *Cx3cr1*^*GFP/GFP*^ MPs overexpress CCL2, which in turn leads to increased CCR2^+^ Mos recruitment from the blood. This in part explains the accumulation of MPs in *Cx3cr1*-deficient mice (Sennlaub *et al*, 2013). Local injections of the traceable nucleotide EdU in light-challenged *Cx3cr1*^*GFP/GFP*^ mice failed to be incorporated in subretinal MPs, suggesting that *in situ* proliferation does not significantly contribute to the accumulation (Supplementary material of (Sennlaub *et al*, 2013)). To evaluate whether subretinal MPs egress from the subretinal space or undergo apoptosis, we adoptively transferred 12,000 CFSE-stained WT and *Cx3cr1*^*GFP/GFP*^ thioglycollate-elicited peritoneal cells (containing 70% Mϕs) in the subretinal space of WT mice (Fig4A, 12 h) and counted the number of F4/80-expressing Mϕs that co-stained for CFSE on RPE and retinal flatmounts once retinal detachment had subsided (8–12 h). Quantifications show that injected CFSE^+^ Mϕs of both genotypes were cleared from the subretinal space over a period of 4 days, but that *Cx3cr1*^*GFP/GFP*^-Mϕ clearance was significantly slower and that *Cx3cr1*^*GFP/GFP*^-Mϕs subsisted in significantly higher numbers at one and two days (Fig4B). We detected no signs of egress from the subretinal space in WT or *Cx3cr1*^*GFP/GFP*^-Mϕ-injected animals, as no CFSE^+^ cells were observed in the inner retina, choroid, blood, local lymph nodes, lung, liver, or spleen by histology or cytometry (data not shown). However, the nuclei of a large number of subretinal CFSE^+^ Mϕs of both genotypes were TUNEL^+^ (Fig4C, TUNEL stained (red) CFSE^+^ (green) WT-Mϕs) and displayed signs of apoptosis (Fig4C, inset: pyknotic and fragmented nuclei). These results suggest that subretinal Mϕ clearance is predominantly mediated by apoptosis, in accordance with observations of inflammation resolution in peripheral tissue (Gautier *et al*, 2013) and in particular with leukocyte clearance in the context of the subretinal immunosuppressive environment (Streilein *et al*, 2002).

Representative image of a RPE flatmount 12 h after the subretinal injection (red marking) of 4 μl PBS with 12,000 CFSE-stained thioglycollate-elicited peritoneal cells that contain 70% macrophages (inset close-up view).

Quantifications of CFSE^+^F4/80^+^ macrophages at different time points after subretinal injections of C57BL/6J and *Cx3cr1*^*GFP*^^/^^*GFP*^ CFSE^+^ macrophages (*n* = 5/per group (12 h) and *n* = 6/per group thereafter; Mann–Whitney *U*-test, C57BL/6J versus *Cx3cr1*^*GFP*^^/^^*GFP*^: 1 day *n* = 20/group *P < 0.0001; 2 day *n* = 6/group *P = 0.0317).

Representative image of TUNEL/Hoechst double-staining 12 h after subretinal injection of C57BL/6J CFSE^+^ macrophages (experiment repeated three times, inset close-up view).

Representative cytometry images of SSC-A/CFSE and CD11b/F4/80 gated analysis of eye cell suspensions prepared 24 h after the injection of *Cx3cr1*^*GFP*^^*/*^^*GFP*^ CFSE^+^ macrophages and cytometric quantification of eye cell suspensions at 24 h after the injection of C57BL/6J and *Cx3cr1*^*GFP*^^*/*^^*GFP*^ macrophages into C57BL/6J (*n* = 16–20/group; Mann–Whitney *U*-test, *P = 0.0024).

Quantification of subretinal CFSE^+^ cells on RPE and retinal flatmounts 24 h after subretinal injections of CFSE^+^ magnetic-bead-sorted bone marrow-derived monocytes (Mo) from C57BL/6J and *Cx3cr1*^*GFP*^^*/*^^*GFP*^ mice into C57BL/6J mice (*n* = 8–12/group; Mann–Whitney *U*-test, *P = 0.0006).

Quantification of subretinal CFSE^+^ cells on RPE and retinal flatmounts 24 h after subretinal injections of CFSE^+^CD11b FACS-sorted brain microglial cells from C57BL/6J and *Cx3cr1*^*GFP*^^*/*^^*GFP*^ mice into C57BL/6J mice (*n* = 9–12/group; Mann–Whitney *U*-test, *P = 0.0087).

Quantification of subretinal CFSE^+^F4/80^+^ macrophages on RPE and retinal flatmounts 24 h after subretinal injections of CFSE^+^ macrophages from C57BL/6J, *Cx3cr1*^*GFP*^^*/*^^*GFP*^*, Cx3cr1*^*GFP*^^*/*^^*GFP*^*ApoE*^−/−^, and *ApoE*^−/−^ mice into C57BL/6J mice (*n* = 8–12/group; one-way ANOVA/Dunnett test of *Cx3cr1*^*GFP*^^*/*^^*GFP*^ versus any other group *P ≤ 0.0001; Mann–Whitney *U*-test, *Cx3cr1*^*GFP*^^*/*^^*GFP*^ versus *Cx3cr1*^*GFP*^^*/*^^*GFP*^*ApoE*^*−/−*^ *P = 0.0006).

Quantification of subretinal CFSE^+^F4/80^+^ macrophages on RPE and retinal flatmounts 24 h after subretinal injections of C57BL/6J CFSE^+^ macrophages into C57BL/6J and with exogenously added APOE3 at 1, 10, or 100 μg/ml calculated intraocular concentrations (*n* = 6–7/group; one-way ANOVA/Dunnett test: C57BL/6J versus 10 μg *P = 0.0488; C57BL/6J versus 100 μg ^‡^*P* = 0.006. Mann–Whitney *U*-test: C57BL/6J versus 10 μg *P = 0.0012; C57BL/6J versus 100 μg ^‡^*P* = 0.0013). Data information: All primary cells were prepared from male mice; all recipient C57BL/6J mice were male. Mo: monocytes; MC: microglial cells; Mϕ: macrophages; SCC-A: side scatter detector A. Scale bars: 1 mm (A); 50 μm (C).

Cytometric quantification of CFSE^+^F4/80^+^CD11b^+^-Mϕs in eye cell suspensions from injected eyes confirmed that *Cx3cr1*^*GFP/GFP*^-Mϕs were present in significantly higher numbers when compared to WT-Mϕs 1 day after adoptive transfer (Fig4D, the GFP fluorescence did not interfere with the several log stronger CFSE signal in the cytometric analysis). The CFSE fluorescence intensity of the F4/80^+^CD11b^+^-Mϕs in the cytometric analysis was strong and homogeneous (Fig4D), suggesting that CFSE uptake by host cells (which leads to variable CFSE intensities) or proliferation (which leads to cell populations with halved CFSE fluorescence intensity) did not occur to a significant degree. Furthermore, WT- and *Cx3cr1*^*GFP/GFP*^-Mϕs did not reveal differences in proliferation *in vitro* (Supplementary Fig S5), suggesting that fast proliferation of *Cx3cr1*^*GFP/GFP*^-Mϕs does not account for the observed difference in the adoptive transfer experiments.

We next evaluated whether the observed differences were specific to peritoneal Mϕs or shared by MPs of other origins. We adoptively transferred CFSE-labeled, magnetic-bead-sorted bone marrow-derived Mos (∽95% pure, Fig4E) and CD11b FACS-sorted brain MCs (∽95% pure, Fig4F) from WT and *Cx3cr1*^*GFP/GFP*^ mice into the subretinal space of WT mice. As with peritoneal Mϕs, *Cx3cr1-*deficient MPs of both origins were significantly greater in number when counted on retinal and RPE/choroidal flatmounts 1 day after the injections.

To evaluate whether MP APOE expression influences the rate of subretinal MP clearance, we adoptively transferred *Cx3cr1*^*GFP/GFP*^*ApoE*^*−/−*^-Mϕs into WT recipients. Strikingly, the increased resistance to subretinal clearance of *Cx3cr1*^*GFP/GFP*^-Mϕs was completely eliminated with *Cx3cr1*^*GFP/GFP*^ *ApoE*^−/−^-Mϕs (Fig4G). Furthermore, exogenous lipid-free APOE3, the predominant human APOE isoform, was sufficient to increase resistance to subretinal clearance when added to WT-CFSE^+^-Mϕs (Fig4H).

Taken together, our results show that *Cx3cr1*-deficient MPs of all origins studied (peritoneum, bone marrow, and brain) are more resistant to subretinal clearance. We show that this increase of resistance to clearance is APOE dependent and that local, recombinant APOE is sufficient to inhibit WT-Mϕs elimination from the subretinal space.

### FAS-FASL signaling mediates subretinal MP clearance

**Figure 5:**
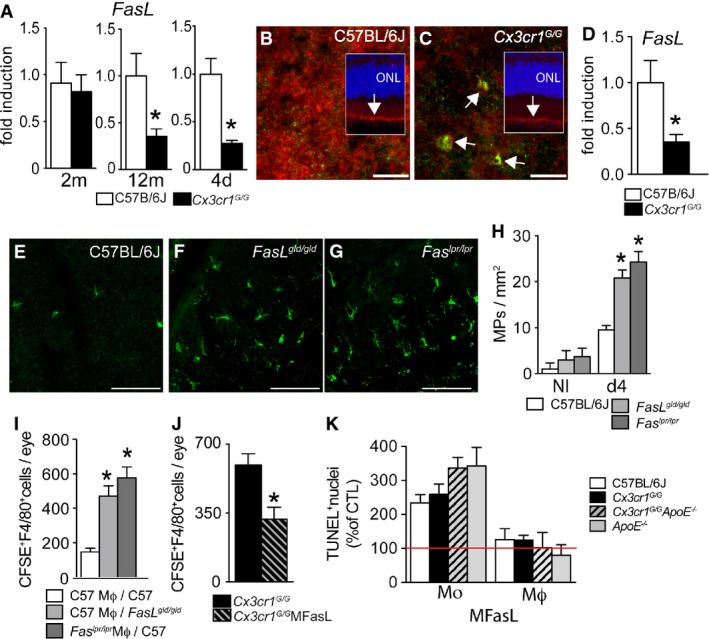
FAS-FASL signaling mediates subretinal MP clearance

The RPE constitutively expresses FASL (CD95L), which in part mediates its immunosuppressiveness (Wenkel & Streilein, 2000). To test whether an alteration of the RPE immunosuppressive environment is associated with subretinal *Cx3cr1*^*GFP/GFP*^-MP accumulation, we first analyzed FasL expression *in vivo*. *FasL* mRNA was similarly expressed in 2-month-old WT and *Cx3cr1*^*GFP/GFP*^ mouse RPE/choroidal plexus, before significant subretinal MP accumulation occurred in *Cx3cr1*^*GFP/GFP*^ mice. In contrast, 12-month-old *Cx3cr1*^*GFP/GFP*^ mice and 2-month-old light-challenged *Cx3cr1*^*GFP/GFP*^ mice with subretinal MP accumulation expressed significantly less *FasL* mRNA (Fig5A, RT–PCR) as compared to WT. Immunohistochemistry on retinal sections and RPE flatmounts of WT and *Cx3cr1*^*GFP/GFP*^ mice (Fig5B and C, FasL, red; IBA-1, green) seemed to confirm the diminished FASL expression in the RPE of *Cx3cr1*^*GFP/GFP*^ mice at 12 months. These results might suggest that *Cx3cr1*-deficient MPs somehow inhibit RPE *FasL* transcription. Indeed, when we injected *Cx3cr1*^*GFP/GFP*^-Mϕs into the subretinal space of WT mice, *FasL* transcription on RPE/choroidal extracts was significantly inhibited after 3 h, when compared to WT-Mϕs-injected eyes (Fig5D, RT–PCR).

Quantitative RT–PCR of *FasL*mRNA normalized with *β-actin*mRNA of 2-month-, 12-month-, and 2-month-old mice after 4 days of light challenge of C57BL/6 and *Cx3cr1*^*GFP*^^*/*^^*GFP*^ mouse RPE/choroid plexus (aging: *n* = 5–6/group; Mann–Whitney *U*-test: 12 months *P = 0.0129; 4 days light challenge: *n* = 7–8/group; *P = 0.029).

Immunohistochemistry of FASL (red) and IBA-1 (green) of a RPE flatmounts and sections (insets, Hoechst staining blue) from a 12-month-old WT (B) and *Cx3cr1*^*GFP*^^*/*^^*GFP*^(C) mouse (representative of three independent experiments, immunostainings omitting the primary antibody served as negative controls).

Quantitative RT–PCR of *FasL*mRNA normalized with *β*-*actin*mRNA of 12-month-old C57BL/6 RPE/choroid plexus extracts 3 h after subretinal injection of C57BL/6J macrophages or *Cx3cr1*^*GFP*^^*/*^^*GFP*^ macrophages (*n* = 6 per group), Mann–Whitney *U*-test, *P = 0.0087.

IBA-1-stained RPE flatmounts of 2-month-old C57BL/6J, *Fasl*^*gld/gld*^, and *Fas*^*lpr/lpr*^ mice after 4 days of light challenge.

Quantification of subretinal IBA-1^+^ mononuclear phagocytes in control (left) and 4-day light-challenged (right) 2-month-old mice of the indicated strains (*n* = 6–10/group ANOVA/Dunnett test at 4-day light challenge: C57BL/6J versus *FasL*^*gld/gld*^ and C57BL/6J versus *Fas*^*lpr/lpr*^ both *P < 0.0001; Mann–Whitney *U*-test at 4-day light challenge: C57BL/6J versus *FasL*^*gld/gld*^ *P < 0.0001; C57BL/6J versus *Fas*^*lpr/lpr*^ *P < 0.0001).

Quantification of subretinal CFSE^+^F4/80^+^ macrophages on RPE and retinal flatmounts 24 h after subretinal injection of C57BL/6J CFSE^+^ macrophages into C57BL/6J and *FasL*^*gld/gld*^ mice and *Fas*^*lpr/lpr*^ macrophages into C57BL/6J mice (*n* = 10–15/group, one-way ANOVA/Dunnett test: C57BL/6J macrophages inj. into C57BL/6J mice versus C57BL/6J macrophages inj. into *Fas*^*gld/gld*^ mice *P = 0.0002; C57BL/6J macrophages inj. into C57BL/6J mice versus *Fas*^*lpr/lpr*^ macrophages inj. into C57BL/6J mice *P < 0.0001. Mann–Whitney *U*-test: C57BL/6J macrophages inj. into C57BL/6J mice versus C57BL/6J macrophages inj. into *FasL*^*gld/gld*^ mice *P < 0.0001; C57BL/6J macrophages inj. into C57BL/6J mice versus *Fas*^*lpr/lpr*^ macrophages inj. into C57BL/6J mice *P < 0.0001).

Quantification of subretinal CFSE^+^F4/80^+^ macrophages on RPE and retinal flatmounts 24 h after subretinal injection of *Cx3cr1*^*GFP*^^*/*^^*GFP*^ CFSE^+^ macrophages into C57BL/6J with or without the Fas agonist MegaFasL (calculated intraocular concentrations 1 ng/ml; *n* = 7–8; Mann–Whitney *U*-test: *P = 0.014).

*In vitro* MegaFasL-induced apoptosis of monocytes and macrophages of the indicated genotypes cultured for 24 h. TUNEL^+^ nucleus quantification expressed as percentage of non-MegaFasL-exposed control. Data information: All primary cells were prepared from male mice; all recipient C57BL/6J or *FasL*^*gld/gld*^ mice were male. Mo: monocytes; MΦ: macrophages. Scale bars: 20 μm (B, C); 50 μm (E–G).

To evaluate whether FAS-FASL signaling participates in MP clearance, we first compared subretinal MP numbers in light-challenged WT (Fig5E), FASL-defective (*FasL*^*gld/gld*^ mice*,* Fig5F), and FAS-defective (*Fas*^*lpr/lpr*^ mice, Fig5G) mice (*FasL*^*gld/gld*^ and *Fas*^*lpr/lpr*^ mice develop lymphadenopathy and systemic autoimmune disease with age, making it difficult to evaluate age-dependent MP accumulation at 12 months). Quantification of subretinal IBA-1^+^ MPs on retinal and RPE/choroidal flatmounts revealed a significant increase of subretinal MPs in 2-month-old *FasL*^*gld/gld*^ and *Fas*^*lpr/lpr*^ mice induced by 4 days of light challenge (Fig5H), similar to that of *Cx3cr1*^*GFP/GFP*^ mice (Fig3). Moreover, adoptive transfer experiments in which we subretinally injected thioglycollate-elicited WT-CFSE^+^-Mϕs into WT or FasL-defective mice (*FasL*^*gld/gld*^ *mice*), and Fas-defective CFSE^+^ Mϕs (prepared from thioglycollate-elicited peritonitis of *Fas*^*lpr/lpr*^ mice) into WT mice, revealed that subretinal CFSE^+^F4/80^+^-Mϕs were significantly greater in number 24 h after the injection when FAS or FASL function was impaired (Fig5I) and comparable to the phenotype observed in *Cx3cr1*^*GFP/GFP*^ Mϕs (Fig3). In addition, co-administration of FAS agonist MegaFasL (Greaney *et al*, 2006) to *Cx3cr1*^*GFP/GFP*^ Mϕs efficiently compensated for the observed FasL downregulation (Fig5D) and significantly reduced the number of subretinal *Cx3cr1*^*GFP/GFP*^ CFSE^+^F4/80^+^-Mϕs after adoptive transfer (Fig5J).

To test whether differences in the susceptibility to FASL-induced MP death might contribute to the protective effect of *ApoE* deletion in subretinal MP accumulation, we exposed Mos and thioglycollate-elicited Mϕs from the different mouse strains to MegaFasL and quantified TUNEL^+^ cells at 24 h *in vitro* (Fig5K). Our results confirm previous reports that FASL is sufficient to induce Mos apoptosis *in vitro* in comparison with Mϕs, which are rather resistant to FASL-induced apoptosis *in vitro* (Um *et al*, 1996; Kiener *et al*, 1997; Park *et al*, 2003). We did not observe a difference between wild-type and *Cx3cr1*^*GFP/GFP*^ cells of either Mos or Mϕs, but a tendency toward increased susceptibility in Mos of both *Cx3cr1*^*GFP/GFP*^ *ApoE*^−/−^ *and ApoE*^−/−^ cells, which might contribute toward the differences in clearance observed *in vivo* (Fig3). These results also highlight that FASL acts along with other factors *in vivo* to induce Mϕ apoptosis in the subretinal space, as the effect of MegaFasL on subretinal clearance of adoptively transferred peritoneal Mϕs (Fig5J) was much stronger than MegaFasL-induced apoptosis *in vitro* (Fig5K). Similarly, a synergistic effect of FasL with other RPE-derived factors has been suggested by the observations that FASL is necessary to eliminate T cells and Mϕs and prevent RPE allograft rejection (Wenkel & Streilein, 2000), but FASL over-expression alone in an allograft, such as a Langerhans cell graft, is not sufficient to prevent rejection (Kang *et al*, 1997).

Taken together, our data show that FAS-FASL signaling is implicated in subretinal MP clearance, that subretinal *Cx3cr1*^*GFP/GFP*^ MPs are associated with a downregulation of RPE FASL expression, and that substitution by MegaFasL restores, in part, the clearance of subretinal *Cx3cr1*^*GFP/GFP*^ MPs.

### APOE promotes subretinal macrophage survival via IL-6

**Figure 6:**
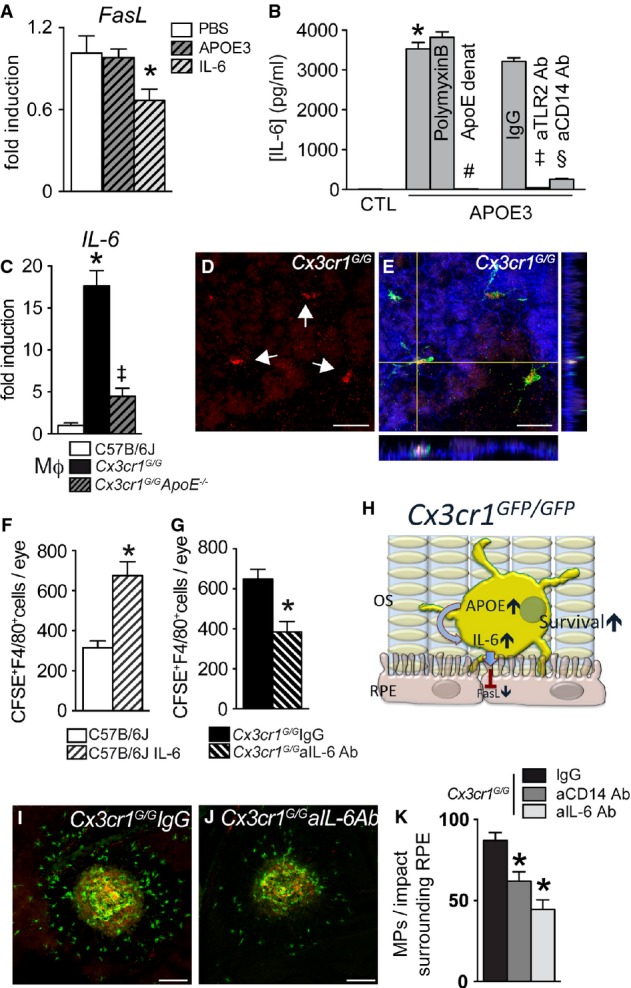
APOE promotes subretinal macrophage survival via IL-6

Our results show that the increased survival of *Cx3cr1*^*GFP/GFP*^-Mϕs is associated with diminished RPE *FasL* transcription, which plays a role in subretinal MP clearance (Fig4). Furthermore, APOE, over-expressed by *Cx3cr1*^*GFP/GFP*^-Mϕs or exogenously added to WT-Mϕs, increases subretinal Mϕs survival (Fig3). However, subretinal injections of recombinant APOE without Mϕs (at a concentration that increases subretinal MP survival (Fig3H) and compared to PBS-injected eyes) did not replicate the observed *FasL* downregulation in RPE/choroid (Fig6A, RT–PCR 3 h after injection), which we observed after adoptive transfer of *Cx3cr1*^*GFP/GFP*^-Mϕs (Fig5D). These results suggest that APOE does not directly influence RPE *FasL* expression. However, recombinant IL-6, shown to downregulate *FasL* expression in lymphocytes (Ayroldi *et al*, 1998), was sufficient to significantly inhibit RPE *FasL* transcription in this experimental setting (Fig6A). APOE and APOA-I can both activate the CD14-dependent innate immunity receptor cluster that contains TLR-2 and TLR-4 in the absence of TLR ligands (Smoak *et al*, 2010). This activation has been shown to induce IL-6, among other cytokines, in the case of APOA-I. Similarly, when we incubated WT-peritoneal-Mϕs with recombinant lipid-free APOE3 for 24 h, IL-6 was very significantly induced (Fig6B). The LPS inhibitor polymyxin B did not inhibit the induction, while 90-min heat denaturation abolished the induction, confirming that LPS contamination of APOE3 is not accountable for the effect, as shown for APOA-I using multiple approaches (Smoak *et al*, 2010). As previously shown for APOA-I, this induction was largely CD14 and TLR2 dependent, as neutralizing antibodies inhibited this effect, when compared to a control IgG (Fig6B). Correspondingly, *Cx3cr1*^*GFP/GFP*^-Mϕs expressed significantly higher amounts of *IL-6* mRNA when compared to WT-Mϕs cultured for 24 h. This effect was significantly inhibited in *Cx3cr1*^*GFP/GFP*^ *ApoE*^−/−^*-*Mϕs (Fig6C), confirming the involvement of APOE. Although *IL-6* was not detectable by RT–PCR in whole-eye mRNA extracts *in vivo*, IL-6 staining (Fig6D, red) was reproducibly detected in subretinal IBA-1^+^-MPs (Fig6E, green) adjacent to the phalloidin^+^RPE (Fig6E, blue, orthogonal, and lateral Z-stack projections of a confocal microscopy picture stack) of 12-month-old *Cx3cr1*^*GFP/GFP*^ mice and light-challenged *Cx3cr1*^*GFP/GFP*^ mice (data not shown).

Quantitative RT–PCR of *FasL*mRNA normalized with *β-actin*mRNA of 12-month-old C57BL/6 RPE/choroid plexus 3 h after subretinal injection of PBS (PBS; *n* = 36), IL-6 (*n* = 17), and APOE3 (*n* = 21); calculated intraocular concentrations: 5 ng/ml and 10 μg/ml, respectively; one-way ANOVA/Dunnett *post hoc* test PBS versus IL-6 *P = 0.038; *t*-test PBS versus IL-6 *P = 0.043.

Mouse IL-6 ELISA of supernatants from C57BL/6J peritoneal macrophages incubated for 24 h in control medium, lipid-free APOE3 (5 μg/ml), APOE3 (5 μg/ml), and polymyxin B (25 μg/ml), heat-denatured APOE3 (dAPOE3, 5 μg/ml), APOE3 (5 μg/ml), and rat IgG1 isotype control (IgG, 100 μg/ml) or APOE3 (5 μg/ml) and rat anti-CD14 antibody (aCD14 Ab, 100 μg/ml) or APOE3 (5 μg/ml) and rat anti-TLR2 antibody (aTLR2 Ab, 100 μg/ml) (*n* = 5–6/group; one-way ANOVA/Bonferroni multi-comparison tests: APOE3 versus CTL *P < 0.0001; dAPOE3 versus APOE3 ^#^*P* < 0.0001; APOE3 IgG versus CTL *P* < 0.0001; APOE3 IgG versus APOE3 aCD14 Ab ^§^*P* < 0.0001; APOE3 IgG versus APOE3 aTLR2 Ab ^++^*P* <0.0001. Mann–Whitney *U*-test: APOE3 versus CTL *P = 0.0043; dAPOE3 versus APOE3 ^#^*P* = 0.0117; APOE3 IgG versus CTL *P* = 0.0080; APOE3 IgG versus APOE3 aCD14 Ab ^§^*P* = 0.0117; APOE3 IgG versus APOE3 aTLR2 Ab ^++^*P* = 0.0079. The experiment was repeated twice with similar results).

Quantitative RT–PCR of *IL-6*mRNA normalized with *S26*mRNA of C57BL/6J and *Cx3cr1*^*GFP*^^*/*^^*GFP*^, and *Cx3cr1*^*GFP*^^*/*^^*GFP*^ *ApoE*^*−/−*^ peritoneal macrophages cultured for 24 h with CX3CL1 (*n* = 5 per group, one-way ANOVA/Bonferroni *post hoc* multi-comparison tests: C57BL/6J versus *Cx3cr1*^*GFP*^^*/*^^*GFP*^ *P < 0.0001 and *Cx3cr1*^*GFP*^^*/*^^*GFP*^ versus *Cx3cr1*^*GFP*^^*/GFP*^ *ApoE*^*−/−*^^‡^*P* < 0.0001. Mann–Whitney *U*-test: C57BL/6J versus *Cx3cr1*^*GFP*^^*/*^^*GFP*^ *P = 0.0079 and *Cx3cr1*^*GFP*^^*/*^^*GFP*^ versus *Cx3cr1*^*GFP*^^*/*^^*GFP*^ *ApoE*^*−/−*^^‡^*P* = 0.0079. The experiment was repeated twice with similar results.).

Orthogonal and lateral Z-stack projection of IL-6 (D, red), IBA-1^+^ (E, green), and phalloidin (E, blue) of IBA^+^ mononuclear phagocytes adjacent to the phalloidin^+^ RPE of a retinal flatmount from a 12-month-old *Cx3cr1*^*GFP*^^*/*^^*GFP*^ mouse. (Representative of three independent experiments, immunostainings omitting the primary antibody served as negative controls).

Quantification of subretinal CFSE^+^F4/80^+^ macrophages on RPE and retinal flatmounts 24 h after subretinal injection of C57BL/6J CFSE^+^ macrophages into C57BL/6J with or without IL-6 (n = 15-20/group; calculated intraocular concentrations of 5 ng/ml. Mann–Whitney *U*-test: *P < 0.0001).

Quantification of subretinal CFSE^+^F4/80^+^ macrophages on RPE and retinal flatmounts 24 h after subretinal injection of *Cx3cr1*^*GFP*^^*/*^^*GFP*^CFSE^+^Mϕs into C57BL/6J with control (IgG, *n* = 16) or anti-IL-6 antibody (aIL-6 Ab, *n* = 8; calculated intraocular concentrations 5 μg/ml; per group. Mann–Whitney *U*-test: *P = 0.0036).

Graphical summary.

7 day laser-injured IBA-1 (green) and CD102 (red) double-stained RPE flatmounts of control IgG- (I) and anti-IL-6-treated (J) *Cx3cr1*^*GFP*^^*/*^^*GFP*^ mice.

Quantification of subretinal IBA-1^+^ mononuclear phagocytes/impact localized on the lesion surrounding RPE of *Cx3cr1*^*GFP*^^*/*^^*GFP*^ mice treated with control IgG, IL-6-, or CD14-blocking antibodies (calculated intraocular concentration 5 μg/ml; *n* = 13–14/group, one-way ANOVA/Dunnett's *post hoc* tests of IgG versus any other group *P < 0.001. Mann–Whitney *U*-test IgG versus anti-IL-6 *P = 0.0021; IgG versus anti CD14 ^*^P = 0.0028). Data information: All primary cells were prepared from male mice; all recipient C57BL/6J mice were male; ONL: outer nuclear layer; OS: outer segments; RPE: retinal pigment epithelium. Scale bars: 20 μm (D, E); 50 μm (I, J).

In addition, recombinant IL-6 added to WT-CFSE^+^Mϕs more than doubled the number of subretinal CFSE^+^F4/80^+^-Mϕs (Fig6F) and an IL-6-blocking antibody significantly decreased subretinal *Cx3cr1*^*GFP/GFP*^CFSE^+^F4/80^+^-Mϕs (Fig6G) 24 h after injection when compared to their controls.

When taken together, the results presented in Figs2, 3, 4 and 5 suggest the following mechanism: *Cx3cr1*^*GFP/GFP*^ MPs express increased amounts of APOE. APOE induces the expression of IL-6 in MPs, which in turn downregulates *FasL* transcription in the RPE. The diminished FASL expression participates in the increased survival time of subretinal *Cx3cr1*^*GFP/GFP*^ MPs (Fig6H).

Finally, to test whether CD14-dependent IL-6 induction does indeed participate in subretinal MP accumulation *in vivo*, we injected control IgG (Fig6I), an IL-6- (Fig6J) or CD14-neutralizing antibody into the vitreous of *Cx3cr1*^*GFP/GFP*^ mice and induced subretinal inflammation with a laser injury (which also facilitates antibody penetration into the subretinal space). Our results show that the accumulation of subretinal IBA-1^+^ MPs (green staining) observed on the RPE adjacent to CD102^+^ CNV (red staining), 7 days after a laser impact, was significantly inhibited when CD14 or IL-6 was neutralized, as was the associated CNV (Supplementary Fig S6).

## Discussion

MPs are activated and accumulate in wet AMD (Oh *et al*, 1999) and around the atrophic lesions of GA adjacent to the RPE (Combadiere *et al*, 2007; Sennlaub *et al*, 2013). Our findings of numerous subretinal MPs in and around large drusen and GA lesions illustrate the significant focal inflammation that is present in AMD. The close contact of subretinal MPs and the RPE suggests that the immunosuppressive properties of the RPE (Streilein *et al*, 2002) are altered in the disease. We show that subretinal MPs, in addition to the RPE, express high levels of APOE similar to MPs in other inflammatory conditions (Rosenfeld *et al*, 1993).

Using *Cx3cr1*^*GFP/GFP*^ mice as a model of subretinal inflammation, we show that increased expression of APOE, observed in *Cx3cr1*^*GFP/GFP*^ MPs, is associated with significant acute (laser- and light-induced) and chronic (age-dependent) subretinal MP accumulation. The observation that *Cx3cr1*-deficient Mϕs are cleared less efficiently from the subretinal space of WT mice suggests that the *in vivo* accumulation is at least in part due to the capacity of *Cx3cr1*-deficient Mϕs to inhibit the RPE immunosuppression and prolong their survival when compared to WT-Mϕs. Using *Cx3cr1*^*GFP/GFP*^ *ApoE*^−/−^-Mϕs and recombinant APOE, we show that this capacity is APOE dependent. We show that APOE increases the expression of IL-6 in a CD14-dependent manner, likely activating the innate immunity receptor cluster, as shown for APOA-I (Smoak *et al*, 2010). While our adoptive transfer experiments suggest that MP-derived APOE is sufficient to induce this effect (all experiments were conducted with C57BL/6J recipients), we do not exclude that RPE-derived APOE participates in the effect *in vivo*, in particular in human AMD where RPE APOE expression was strong. IL-6 in turn inhibited MP elimination, as a CD14- and IL-6-blocking antibody partly reversed the elimination deficit of adoptively transferred Mϕs and inhibited subretinal MP accumulation in a model of laser-induced inflammation in *Cx3cr1*^*GFP/GFP*^ mice. While we observe that extracellular recombinant APOE can induce IL-6, we do not exclude the possibility that intracellular APOE participates in the increased IL-6 secretion observed in *Cx3cr1*^*GFP/GFP*^ MPs. IL-6 secreted from the subretinal MPs in turn downregulates *FasL* transcription in RPE. Using light-induced subretinal inflammation and adoptive transfer experiments and *FasL*^*gld*^ and *Fas*^*lpr*^ mice, we show that FASL/FAS signaling takes part in MP elimination. Indeed, a pharmacological FAS agonist was able to partly reverse the elimination deficit of adoptively transferred *Cx3cr1*-deficient Mϕs, similar to the IL-6-blocking antibody. Interestingly, *ApoE*-deficient monocytes, but not macrophages, also showed a tendency toward increased susceptibility to FASL-induced apoptosis *in vitro*, which might contribute toward the differences in clearance observed *in vivo*.

The accumulation of subretinal MPs is likely the result of MP recruitment that exceeds the MP elimination rate. In the present study, we have concentrated on MP elimination, but we do not suggest that increased recruitment is less important. Indeed, APOE like APOA-I also increases CCL2 expression in MPs, and we have previously shown the role of CCL2 in subretinal MP accumulation (Sennlaub *et al*, 2013). Also, our study focused on FasL expression, but other RPE-derived signals likely play a role in MP elimination and non-transcriptional interactions such as proteolytic cleavage of FASL probably participate in the mechanism.

The cross talk of APOE and APOA-I, the reverse cholesterol transport (RCT), and innate immunity are complex. On the one hand, excess of cholesterol and cholesterol crystals can activate Toll-like receptors (TLR) and APOE and APOA-I can bind and neutralize TLR ligands and inhibit the induction of inflammatory cytokines (Azzam & Fessler, 2012). On the other hand, Smoak *et al* demonstrated that APOE and APOA-I are capable of triggering TLR2/4 signaling in the absence of TLR ligands, possibly by extracting cholesterol from lipid rafts in which the CD14-containing innate immunity receptor cluster (necessary for TLR2/4 signaling) is located (Smoak *et al*, 2010). Our results using a CD14-blocking antibody and a recent report that subretinal MP accumulation is TLR4 dependent in a retinal degeneration model (Kohno *et al*, 2013) back the notion that TLR signaling is involved in the alteration of the immunosuppressive environment. The involvement of reverse cholesterol transport (RCT) in AMD might also be supported by the recent observation that APOA-I levels are elevated in the vitreous of AMD patients (Koss *et al*, 2014). Furthermore, a polymorphism of the ATP binding cassette transporter 1 (ABCA1, associated with low HDL and therefore possibly impaired RCT) has recently been shown to be protective against advanced AMD (Chen *et al*, 2010). Moreover, IL-6 levels are associated with early AMD incidence (Klein *et al*, 2014) and late AMD (Seddon *et al*, 2005; Klein *et al*, 2008) and might participate in the weakening of RPE immunosuppression in AMD.

*In vitro*, the RPE has been shown to secrete APOE preferentially to its basal side (Ishida *et al*, 2004). Our immunohistochemical localization to the basal side of the RPE in healthy human donor tissue might suggest that this is also the case *in vivo*. Interestingly, this polarization of the APOE signal was lost in GA donor tissue, where a strong APOE signal was observed throughout the RPE cells adjacent to the lesion. Apical APOE release from RPE into the subretinal space might participate in increasing its subretinal concentration in addition to APOE released from subretinal MPs.

It has been suggested that a lack of APOE participates in both the accumulation of lipids and the drusen formation (Ong *et al*, 2001; Malek *et al*, 2005; Johnson *et al*, 2011). Indeed, *ApoE*^−/−^- and *APOE4* mice fed on a high-fat diet develop lipid accumulations in the Bruch's membrane, which has been proposed as similar to early AMD (Ong *et al*, 2001; Malek *et al*, 2005). While these observations might apply to early AMD, they are unlikely to play a role in late AMD in which increased APOE immunoreactivity is observed (Klaver *et al*, 1998; Anderson *et al*, 2001) and in which the *APOE4-*allele plays a protective role (McKay *et al*, 2011).

Taken together, the results of our study describe a new cellular and molecular mechanism that may participate in the weakened subretinal immunosuppression and chronic inflammation of AMD. Although we have detected this mechanism in mice, increased APOA-I levels in the vitreous of AMD patients (Koss *et al*, 2014) and elevated IL-6 levels with AMD suggest that a similar mechanism is at work in human AMD patients. In the future, the inhibition of IL-6 and/or the restoration of RPE immunosuppression might be employed to control pathologic subretinal inflammation in AMD.

## Materials and Methods

### APOE, IBA-1, and CD18 immunohistochemistry on donor samples

Donor eyes with a known history of AMD and controls were collected through the Minnesota Lions Eye bank. Informed consent was obtained for all donor eyes by the Minnesota Lions Eye bank, and the experiments conformed to the principles set out in the WMA Declaration of Helsinki. Postmortem fundus photographs were taken, and the posterior segment was fixed 4 h in 4% PFA, transported in PBS, dissected, imbedded in paraffin, and sectioned (five control maculae; five GA donor maculae). Donors gave informed consent in accordance with the eye bank's ethics committee. Five tonsillectomy surgical samples, removed for recurrent acute tonsillitis, were recuperated from tonsillectomies at the Fondation Rothschild and then fixed and sectioned in the same way. For flatmount immunohistochemistry, donor eyes with visible atrophic areas (five eyes), visible large drusen on RPE flatmounts (five eyes), and controls (three eyes) were dissected into approximately 5 × 5 mm tissue parts, and immunohistochemistry was performed on submerged samples. APOE (M068-3 mouse anti-human, citrate buffer heat antigen retrieval for paraffin sections, MBL), IBA-1 (rabbit anti-human, citrate buffer heat antigen retrieval, Wako Chemicals), and CD18 (MCA503, rat anti-human, citrate buffer heat antigen retrieval, Abd Serotec) immunohistochemical analyses were performed using appropriate fluorescent or alkaline phosphatase-coupled secondary antibodies (Molecular Probe) using a Fast Red substrate kit (Sigma).

### Animals

Wild-type and *Cx3cr1*^*GFP/GFP*^, *ApoE*^−/−^, *Fas*^*lpr*^, and *FasL*^*gld*^ were purchased (Charles River Laboratories, Jackson laboratories), and *Cx3cr1*^*GFP/GFP*^ *ApoE*^−/−^ mice were generated. All mice were negative for the *Crb1*^*rd8*^, *Pde6b*^*rd1*^, and *Gnat2*^*cpfl3*^ mutations. Mice were housed in the animal facility under specific pathogen-free condition, in a 12/12 h light/dark (100–500 lx) cycle with water and normal diet food available *ad libitum*. All experimental protocols and procedures were approved by the local animal care ethics committee ‘Comité d’éthique en expérimentation animale Charles Darwin’ (N° p3/2008/54).

### Light challenge and laser injury model

Two- to four-month-old mice were adapted to darkness for 6 h, and pupils dilated and exposed to green LED light (starting at 2AM, 4,500 lx, JP Vezon equipments) for 4 days as previously described (Sennlaub *et al*, 2013). Laser coagulations were performed with a 532-nm ophthalmological laser mounted on an operating microscope (Vitra Laser, 532 nm, 450 mW, 50 ms and 250 μm). Intravitreal injections of 2 μl of PBS, isotype control rat IgG1, rat anti-mouse IL-6 (R&D Systems), and rat anti-mouse CD14 (BD Biosciences) were performed using glass capillaries (Eppendorf) and a microinjector. The 2 μl solution of the antibodies was injected at 50 μg/ml, corresponding to an intraocular concentration of 5 μg/ml assuming their dilution by approximately 1/10 in the intra-ocular volume.

### Immunohistochemistry, MP quantification, and histology

Human and murine RPE and retinal flatmounts and human and murine sections were stained and quantified as previously described (Sennlaub *et al*, 2013) using polyclonal goat anti-human APOE (Millipore), polyclonal rabbit anti-IBA-1 (Wako), polyclonal rabbit anti-rat FASL (Millipore), monoclonal rat anti-mouse IL-6 (R&D Systems), AlexaFluor 555 phalloidin (Mol probes), and rat anti-mouse CD102 (clone 3C4, BD Biosciences Pharmingen) appropriate secondary antibodies and counterstained with Hoechst if indicated. Preparations were observed with fluorescence microscope (DM5500, Leica) or a FV1000 (Olympus) confocal microscope. Histology of mice eyes and photoreceptor quantification were performed as previously described (Sennlaub *et al*, 2013).

### Cell preparations and cell culture

Resident and thioglycollate-elicited peritoneal cells, peritoneal macrophages, bone marrow-derived monocytes, brain microglial cell, and photoreceptor outer segment (POS) isolation, and MP-retinal explant co-cultures (all in serum-free X-VIVO 15 medium) were performed as previously described (Sennlaub *et al*, 2013). In specific experiments, cells were stimulated with recombinant human CX3CL1 or APOE3 (5 μg/ml, Leinco Technologies), APOE3 (5 μg/ml) with polymyxin B (25 μg/ml, Calbiochem), heat-denatured APOE3 (5 μg/ml, 95°C, 90 min), rat anti-IgG isotype control (100 μg/ml, R&D), rat anti-mouse CD14 (100 μg/ml, R&D), mouse anti-mouse TLR2 (100 μg/ml, Invivogen), and POS prepared as previously described (Molday *et al*, 1987). For *in vitro* apoptosis experiments, 100,000 Mos or Mϕs of the different genotypes were cultured for 24 h with or without MegaFasL (1 ng/ml, AdipoGen). TUNEL staining (*In Situ* Cell Death Detection Kit, Roche Diagnostics) was performed according to the manufacturer's instructions; TUNEL^+^ and Hoechst^+^ nuclei were counted automatically using the Array Scan (Thermo Fischer).

### Subretinal mononuclear phagocyte cell clearance

Thioglycollate-elicited peritoneal cells (containing 70% Mϕs), bone marrow-derived monocytes (∽95% pure), and brain microglial cell (∽95% pure) were labeled in 10 μM CFSE (Life Technologies). Cells were washed and resuspended in PBS. 12,000 cells (4 μl) were injected into the subretinal space of anesthetized 2-month-old mice using a microinjector and glass microcapillaries (Eppendorf). A hole was pierced with the glass capillary prior to the subretinal injection to avoid intra-ocular pressure increase and to allow retinal detachment with 4 μl of solution. The subretinal injection was verified by fundoscopy. In specific experiments, Mϕs were co-injected with rhAPOE (APOE3, Leinco Technologies), rmIL-6, rat anti-mouse IL-6, rat anti-mouse CD14, the isotype control rat IgG1 (R&D Systems), or MegaFasL (AdipoGen). Indicated intraocular concentrations were calculated as a dilution of 10× of the injected solution, as the injected 4 μl corresponds to approximately 1/10 of the intraocular volume. Eyes were enucleated after 24 h, fixed in 4% PFA, and flatmounted. The flatmounts were double-labeled with anti-F4/80 antibody to identify CFSE^+^F4/80^+^Mϕs and counted on the subretinal aspect of the retinal flatmount and the RPE/choroid flatmount of each eye. Eyes with subretinal hemorrhages were discarded. Double-labeled MPs in subretinal space were quantified on RPE flatmounts and the subretinal side of retinal flatmounts.

### Flow cytometry

Cytometry was performed as previously described (Camelo *et al*, 2012), using anti-CD11b PE, anti F4/80 Pacific Blue, or APC, streptavidin APC (all from Abd Serotec). Acquisition was performed on LSRII cytometer (BD Biosciences), and data were analyzed with FlowJo 7.9.

### Western blot, reverse transcription and real-time polymerase chain reaction, and ELISA

WB analysis was performed using a polyclonal goat anti-ApoE (Millipore) and a polyclonal goat anti-Mertk (R&D) as previously described (Houssier *et al*, 2008). RT–PCRs using SYBR Green (Life Technologies) and ELISAs using mouse IL-6 DuoSet (R&D Systems) were performed as previously described (Sennlaub *et al*, 2013).

### Terminal deoxynucleotidyl transferase dUTP nick end labeling (TUNEL) on flatmounts

4% PFA-fixed retinal flatmounts were post-fixed in frozen methanol/acetic acid (2:1) for 30 min and washed in PBS. Flatmounts were incubated overnight at 4°C with the terminal transferase and the supplied buffer (*In Situ* Cell Death Detection Kit, Roche Diagnostics). Flatmounts were then incubated at 37°C for 90 min, and the reaction was stopped by washing with PBS. Nuclei were counterstained with Hoechst (Sigma-Aldrich). Flatmounts images were captured with a DM5500 microscope (Leica).

### Statistical analysis

Sample sizes for our experiments were determined according to our previous studies and a pilot study concerning subretinal MP injections. The pilot study revealed that severe hemorrhage secondary to subretinal injection interferes with MP clearance and was used as exclusion criteria. Graph Pad Prism 6 (GraphPad Software) was used for data analysis and graphic representation. All values are reported as mean ±SEM. Statistical analysis was performed by one-way ANOVA followed by Bonferroni or Dunnett's post-test (multiple comparison) or Mann–Whitney *U*-test (2-group experiments) for comparison among means depending on the experimental design. The *n* and *P*-values are indicated in the figure legends.

## The paper explained

### Problem

Physiologically, the subretinal space, located between the retinal pigment epithelium (RPE) and the photoreceptors, is a zone of immune privilege mediated in part by immunosuppressive RPE signals that induce apoptosis of leukocytes. In healthy subjects, this space is therefore devoid of all leukocytes, including mononuclear phagocytes (MP), a family of cells that include microglial cells, monocytes, and macrophages among others. Nevertheless, MPs accumulate subretinally in contact with the RPE in age-related macular degeneration (AMD) and likely participate in neurodegeneration and neovascularization in the disease. The reasons for the breakdown of subretinal immuno-suppression and accumulation of MPs in AMD remain unknown.

### Results

This study shows that subretinal MPs in AMD express high amounts of apolipoprotein E (APOE), a lipoprotein that plays a crucial role in reverse cholesterol transport. Using *Cx3cr1*^−/−^ mice that accumulate subretinal MPs with age and after light- or laser-induced stress, we demonstrate that APOE prolongs subretinal MP survival and is necessary for subretinal MP accumulation. We demonstrate that increased APOE induces IL-6 in MPs, via the activation of the TLR2-CD14-dependent innate immune receptor cluster. IL-6 in turn represses RPE FasL expression, prolongs subretinal MP survival, and promotes chronic subretinal inflammation.

### Impact

Taken together, the results of our study describe a new cellular and molecular mechanism that explains the weakening of subretinal immunosuppression and consequently participates in chronic inflammation in AMD. Although detected in mice, the association of the APOE isoform 2, which is associated with higher APOE concentrations and elevated IL-6 serum levels in AMD, suggests that a similar mechanism is at work in human AMD patients. In future, the inhibition of IL-6 and the restoration of RPE immunosuppression could be used to control pathologic subretinal inflammation in AMD.

## Supporting Information

Supplementary Figures

Review Process File
