# Supplementary material for: Apolipoprotein E promotes subretinal mononuclear phagocyte survival and chronic inflammation in age-related macular degeneration
Source: EMBO Mol Med. 2015 Jan 20;7(2):211–26. doi: 10.15252/emmm.201404524 (PMC4328649; doi:10.15252/emmm.201404524)
Supplement: Supplementary file 1 [file emmm0007-0211-sd1.pdf]

Supplementary Figure 1: APOE localization in mice eyes

Immunohistochemical localization of APOE on retinal sections of a 12 month old *WT*-mouse showed APOE localization mainly in the RPE and inner retina as previously described (1) (A and B). Additionally, we detected a strong signal in cells apposed to the RPE on retinal sections in aged *Cx3cr1*$^{GFP/GFP}$-mice (C).

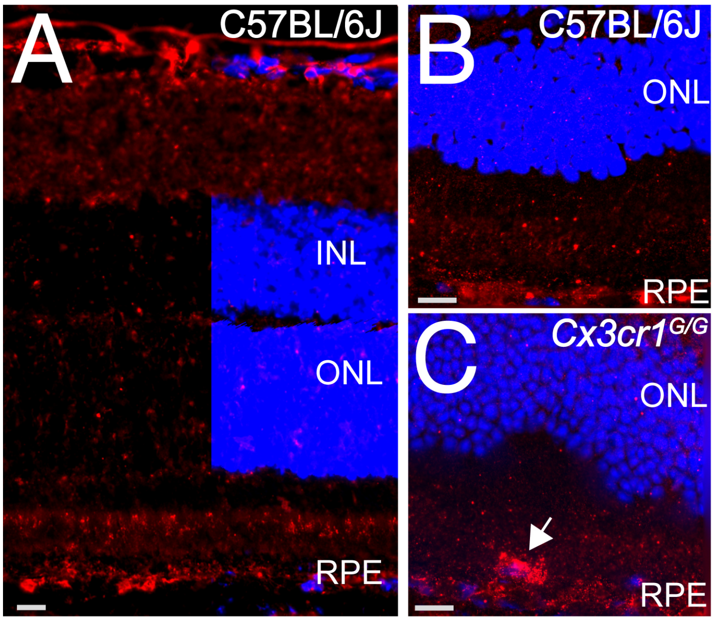

A-C: Immunohistochemistry of APOE (red) and Hoechst (blue) on sections of 12m-old C57BL/6J (A-B) and *Cx3cr1*$^{GFP/GFP}$ (C) mice. Arrow shows a strong APOE reactivity in a subretinal cell apposed to the RPE (representative of 3 independent experiments, immunostaining on *ApoE*$^{-/-}$mice served as negative control). INL: Inner nuclear layer; ONL: Outer nuclear layer ; RPE: Retinal Pigment Epithelium. Scale bars=10µm.

Supplementary Figure 2: *Cx3cr1*$^{GFP/GFP}$*ApoE*$^{-/-}$-mice are significantly protected against age-dependent photoreceptor degeneration observed in *Cx3cr1*$^{GFP/GFP}$-mice. Outer nuclear layer (ONL) containing the photoreceptor nuclei on histological sections of 12m-old *WT*-, *ApoE*$^{-/-}$-, *Cx3cr1*$^{GFP/GFP}$-, and *Cx3cr1*$^{GFP/GFP}$ *ApoE*$^{-/-}$-mice (A-D). At equal distance from the optic nerve, *ApoE*$^{-/-}$-mice presented a thinned ONL, attributed to disturbed systemic lipid transport and retinal cholesterol trafficking as previously described (2). Note that the ONL of *Cx3cr1*$^{GFP/GFP}$*ApoE*$^{-/-}$-mice was similar to *ApoE*$^{-/-}$-mice and thicker and more regular than in *Cx3cr1*$^{GFP/GFP}$-mice. Photoreceptor nuclei row counts (E) and calculation of the area under the curve (F) showed that *Cx3cr1*$^{GFP/GFP}$*ApoE*$^{-/-}$-mice were significantly protected against age-dependent photoreceptor cell loss when compared to *Cx3cr1*$^{GFP/GFP}$-mice and not significantly different from *ApoE*$^{-/-}$-mice.

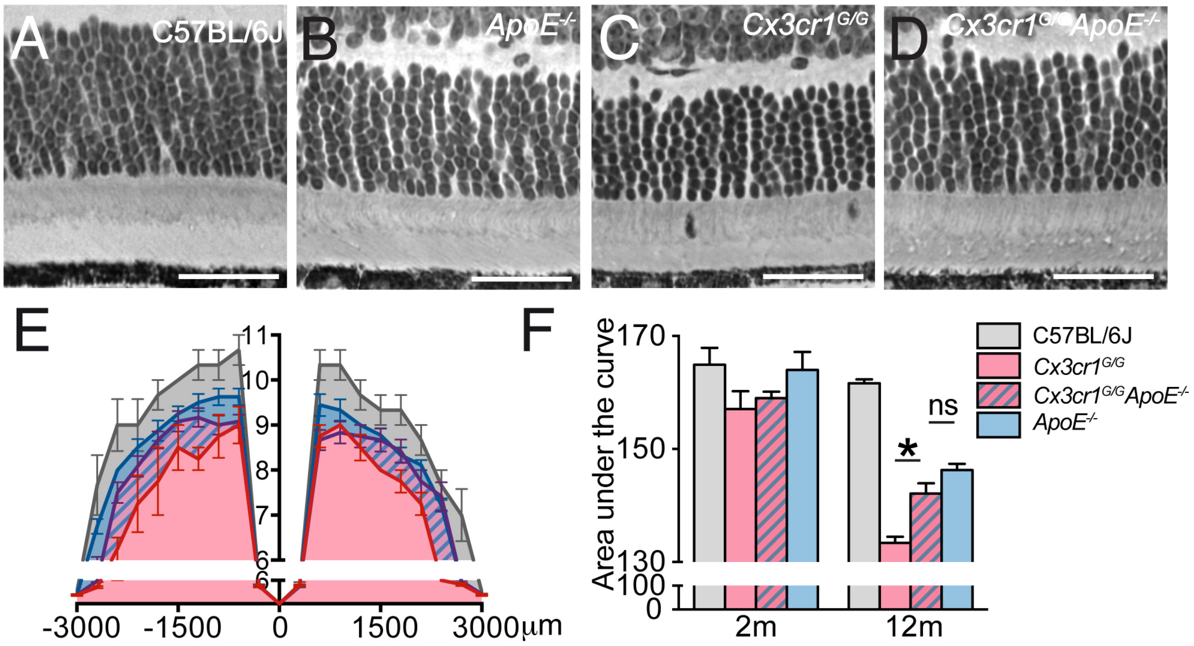

A-D: Micrographs, taken 1000 µm from the optic nerve of 12m-old C57BL/6J (A), *ApoE*$^{-/-}$ (B), *Cx3cr1*$^{GFP/GFP}$(C), and *Cx3cr1*$^{GFP/GFP}$*ApoE*$^{-/-}$mice (D).

E: Photoreceptor nuclei rows at increasing distances (-3000µm: inferior pole, +3000µm: superior pole) from the optic nerve (0µm) in 12m-old C57BL/6J, *Cx3cr1*$^{GFP/GFP}$, *Cx3cr1*$^{GFP/GFP}$*ApoE*$^{-/-}$, and *ApoE*$^{-/-}$ mice.

F: Quantification of the area under the curve of photoreceptor nuclei row counts of 2m- (left) and 12m-old (right) C57BL/6J, *Cx3cr1*$^{GFP/GFP}$, *Cx3cr1*$^{GFP/GFP}$*ApoE*$^{-/-}$, and *ApoE*$^{-/-}$ mice (n=5-12; One-way ANOVA/ Bonferronni at 12m * *Cx3cr1*$^{GFP/GFP}$vs *Cx3cr1*$^{GFP/GFP}$*ApoE*$^{-/-}$ p=0.001 and NS *Cx3cr1*$^{GFP/GFP}$*ApoE*$^{-/-}$ vs *ApoE*$^{-/}$ p=0,3447. Independent Mann & Whitney t tests at 12m of * *Cx3cr1*$^{GFP/GFP}$ vs *Cx3cr1*$^{GFP/GFP}$*ApoE*$^{-/-}$ p=0.0043 and *NS* *Cx3cr1*$^{GFP/GFP}$*ApoE*$^{-/-}$ vs *ApoE*$^{-/-}$ p=1.076) Mice were taken from several (≥3) independent cages for the quantifications. Scale bar A-D = 50µm.

Supplementary Figure 3: *Cx3cr1$^{GFP/GFP}$ApoE$^{-/-}$*-mice are significantly protected against exaggerated CNV observed in *Cx3cr1$^{GFP/GFP}$*-mice.

We previously showed that *Cx3cr1* deletion leads to exaggerated inflammation and CNV after laser-injury (3). Representative CD102-stained RPE flatmounts of *Cx3cr1$^{GFP/GFP}$*-mice (A) show a smaller area of CD102^{+}CNV in *Cx3cr1$^{GFP/GFP}$ ApoE$^{-/-}$*-mice (B) seven days after the laser-injury. Quantification of the CD102 staining in the different strains confirm the exaggerated CNV in *Cx3cr1$^{GFP/GFP}$*-mice and shows that CNVs are significantly smaller in *Cx3cr1$^{GFP/GFP}$ ApoE$^{-/-}$*-mice (C).

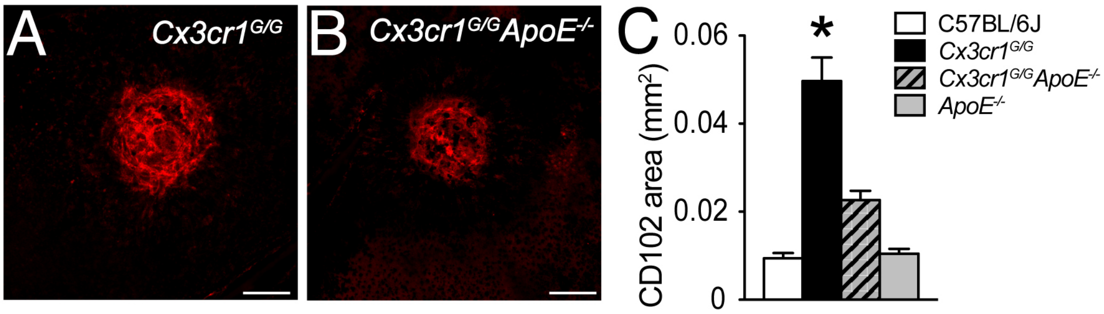

A-B: Immunohistochemistry of CD102 (red) on RPE/choroidal flatmount from 2m-old *Cx3cr1$^{G/G}$* (A) and *Cx3cr1$^{G/G}$ApoE$^{-/-}$* (B) mice, 7 days after laser injury.

C: Quantification of CD102^{+} CNV area on RPE/choroidal flatmounts of C57BL/6J (n=8 eyes), *Cx3cr1$^{GFP/GFP}$* (n=8), *Cx3cr1$^{GFP/GFP}$ApoE$^{-/-}$* (n=10) and *ApoE$^{-/-}$* (n=10) mice, 7 days after laser injury (n=8-10/group; One-way ANOVA/Bonferroni *Cx3cr1$^{GFP/GFP}$* vs *Cx3cr1$^{GFP/GFP}$ApoE$^{-/-}$* * p<0.0001. Independent Mann & Whitney t-tests of *Cx3cr1$^{GFP/GFP}$* vs *Cx3cr1$^{GFP/GFP}$ApoE$^{-/-}$*: p<0.0001). Scale bars = 50µm.

Supplementary Figure 4: subretinal MP accumulation in 12m- old offspring from *Cx3cr1*$^{+/GFP}$ breeders

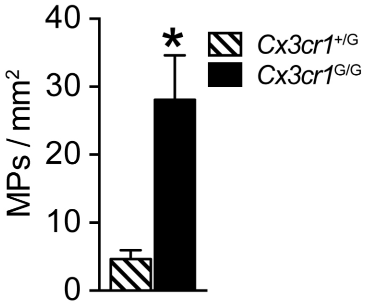

Quantification of subretinal IBA-1^{+}MPs in 12m-old offspring from the same *Cx3cr1*$^{+/GFP}$ breeders (n=5-7/group, Mann & Whitney t test *p=0.0095)

Supplementary Figure 5: Macrophage proliferation *in vitro*

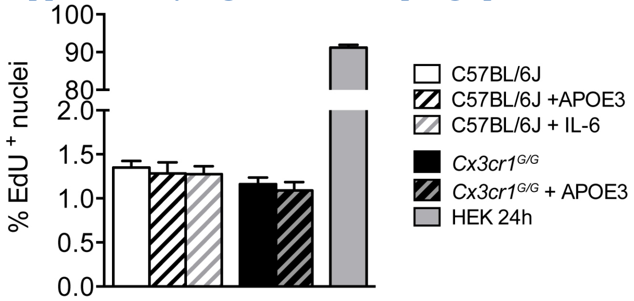

Quantification of traceable nucleotide EdU^{+}nuclei after 1d of cell culture. The proliferation rates of *WT*- and *Cx3cr1*$^{GFP/GFP}$-Mfs were low and not significantly different from each other. APOE3 or IL-6 did not increase the proliferation rate. Neither APOE3 nor IL-6 increased the proliferation rate.

Supplementary Figure 6: anti-CD14, and anti-IL6 antibodies inhibit subretinal choroidal neovascularization compared to IgG control.

Representative CD102-stained RPE flatmounts of IgG control-treated *Cx3cr1*$^{GFP/GFP}$-mice (A) shows a wider area of CD102^{+}CNV than in IL6-blocking antibody-treated *Cx3cr1*$^{GFP/GFP}$-mice (B) seven days after the laser-injury. Quantification of the CD102 staining shows that the CNV in *Cx3cr1*$^{GFP/GFP}$-mice treated with CD14-, and IL-6-blocking antibodies are significantly smaller when compared to CNV in control IgG treated *Cx3cr1*$^{GFP/GFP}$ -mice (C).

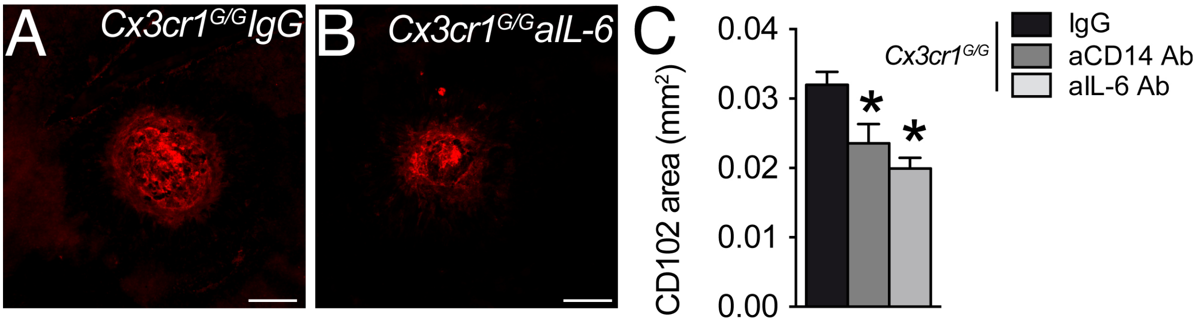

A-B: Immunohistochemistry of CD102 (red) on RPE/choroidal flatmount from 2m-old IgG -treated (A) and IL6-blocking antibody-treated (B) *Cx3cr1*$^{G/G}$ mice, 7 days after laser injury.

C: Quantification of CD102^{+} CNV area on RPE/choroidal flatmounts of of *Cx3cr1*$^{GFP/GFP}$ mice treated with control IgG, IL-6- or CD14- blocking antibodies (calculated intraocular concentration 5µg/ml; n=8-10 eyes/group. One-way ANOVA/Dunnett's post-hoc tests of IgG vs any other group *p=0,0197. Mann & Whitney t test * IgG vs anti IL-6 p<0.0001; IgG vs anti CD14 p=0.015). Scale bars = 50µm.

References for the the supplementary material section

1. Anderson DH, Ozaki S, Nealon M, Neitz J, Mullins RF, Hageman GS, and Johnson LV. Local cellular sources of apolipoprotein E in the human retina and retinal pigmented epithelium: implications for the process of drusen formation. *Am J Ophthalmol.* 2001;131(6):767-81.
2. Ong JM, Zorapapel NC, Rich KA, Wagstaff RE, Lambert RW, Rosenberg SE, Moghaddas F, Pirouzmanesh A, Aoki AM, and Kenney MC. Effects of cholesterol and apolipoprotein E on retinal abnormalities in ApoE-deficient mice. *Invest Ophthalmol Vis Sci.* 2001;42(8):1891-900.
3. Combadiere C, Feumi C, Raoul W, Keller N, Rodero M, Pezard A, Lavalette S, Houssier M, Jonet L, Picard E, et al. CX3CR1-dependent subretinal microglia cell accumulation is associated with cardinal features of age-related macular degeneration. *J Clin Invest.* 2007;117(10):2920-8.
