# Supplementary material for: Apolipoprotein E promotes subretinal mononuclear phagocyte survival and chronic inflammation in age-related macular degeneration
Source: EMBO Mol Med. 2015 Jan 20;7(2):211–26. doi: 10.15252/emmm.201404524 (PMC4328649; doi:10.15252/emmm.201404524)
Supplement: Supplementary file 2 [file emmm0007-0211-sd2.pdf]

Apolipoprotein E promotes subretinal mononuclear phagocyte survival and chronic inflammation in age-related macular degeneration

Olivier Levy, Bertrand Calippe, Sophie Lavalette, Shulong J. Hu, William Raoul, Elisa Dominguez, Michael Housset, Michel Paques, José-Alain Sahel, Alexis-Pierre Bemelmans, Christophe Combadiere, Xavier Guillonneau and Florian Sennlaub

Corresponding author: Florian Sennlaub, Institut de la Vision

Review timeline:

Submission date:	08 August 2014
Editorial Decision:	29 August 2014
Revision received:	24 November 2014
Editorial Decision:	09 December 2014
Revision received:	11 December 2014
Accepted:	15 December 2014

Transaction Report:

(Note: With the exception of the correction of typographical or spelling errors that could be a source of ambiguity, letters and reports are not edited. The original formatting of letters and referee reports may not be reflected in this compilation.)

Editor: Roberto Buccione

1st Editorial Decision 29 August 2014

Thank you for the submission of your manuscript to EMBO Molecular Medicine. We have now heard back from the three Reviewers whom we asked to evaluate your manuscript.

You will see that the Reviewers are supportive of you work, although they do express a number of concerns that prevent us from considering publication at this time. I will not dwell into much detail, as the evaluations are self-explanatory. I would like, however, to highlight a few main points.

Reviewer 1, in addition to mentioning a number of technical and lexical issues that require your action, raises a specific concern with respect to the genetic makeup of the control animals used.

Reviewer 2 would like you to verify how ApoE-deficient and control macrophages undergo SA-induced apoptosis in vitro and also suggests a more detailed analysis of patient material.

Reviewer 3 is also positive albeit more reserved. S/he is not convinced of how the human and mouse data correlate (given the significant differences between mouse and human) and in general of the translational value of the conclusions. This Reviewer also points to the omission of a relevant reference. One important aspect that s/he raises (and I fully agree) is that the manuscript would enormously benefit from substantial streamlining, simplification and reworking, on one hand to make it easier on the reader, on the other to make it more accessible to a non specialist readership (which is very important for EMBO Molecular Medicine). You will note that Reviewer 2 also

mentions this aspect.

Considered all the above, while publication of the paper cannot be considered at this stage, we would be pleased to consider a revised submission, with the understanding that the Reviewers' concerns must be addressed as outlined above, with additional experimental data where appropriate and that acceptance of the manuscript will entail a second round of review.

Please note that it is EMBO Molecular Medicine policy to allow a single round of revision only and that, therefore, acceptance or rejection of the manuscript will depend on the completeness of your responses included in the next, final version of the manuscript.

As you know, EMBO Molecular Medicine has a "scooping protection" policy, whereby similar findings that are published by others during review or revision are not a criterion for rejection. However, I do ask you to get in touch with us after three months if you have not completed your revision, to update us on the status. Please also contact us as soon as possible if similar work is published elsewhere.

I look forward to seeing a revised form of your manuscript as soon as possible.

***** Reviewer's comments *****

Referee #1 (Comments on Novelty/Model System):

Identification of potential therapeutic targets of signaling pathways contributing to the late forms of age-related macular degeneration is paramount. The authors outline the involvement of apolipoprotein E, IL-6 and FasL associated pathways in prolonged survival of mononuclear phagocytes in the sub-retinal space, contributing the disease progression. A concern regarding the mice has been raised to authors and essentially involves clarification of control mice used, to include wt mice (littermate controls) rather than C57BL6J mice (from Jax mice) which may not express the same genetic changes that occur with prolonged breeding to a knockout mouse. As such the changes seen may not be due to more than just knockout of the gene of interest (apoE, Cx3cr1, etc).

Referee #1 (Remarks):

Overall comments:

In this manuscript Levy et al. report on features of subretinal mononuclear phagocytes (MPs), which are reported to infiltrate the subretinal space in AMD patients (by this group and others). They find that subretinal MPs in AMD patients express apoE. In Cx3Cr1-/- mice they confirm their previous finding of increased subretinal MPs, extending it with the observation that these MPs also express high levels of apoE in this genetic model, and that apoE deletion reverses this pathology. Mechanistically they suggest that the infiltration and prolonged survival of these MPs occurs through activation of CD14 and increased expression of IL-6 due to increased apoE expression. Given that the identification of potential therapeutic targets of signaling pathways contributing to the late forms of age-related macular degeneration (especially GA) is an important unmet need, the reported findings of the involvement of apoE, IL6 and FasL associated pathways in prolonged survival of MPs in the subretinal space, are significant additions to the field of AMD research and of high importance.

Specific comments:

1. Introduction-1st paragraph: drusen accumulate between the RPE basal lamina and the inner collagenous layer of Bruch's. As such they are within Bruch's membrane, not "located on the Bruch's membrane". Please correct.
2. Figure 1. The flatmount images provide a very nice overview of the distribution of IBA1+ cells. However, cross-sections would illustrate co-localization of apoE protein within subretinal IBA1+

cells better and is recommended, given this study hinges on the premise that subretinal MPs "strongly express apoE".
3. Panels E-H within the last two paragraphs of the "subretinal MPs accumulate in AMD and express apoE" results section are incorrectly referred to, please correct.
4. Figure 2G. Please provide a control reference for the western blot. Another secreted protein?
5. Provide detailed description of quantification methods used for Figure 2, panels L-N. How far out of the lesion were MPs counted? Etc...
6. Figure 3. What is the quantification measure in the charts? Number of cells/eye? %+/eye? All normalized to C57BL/6J? Please clarify.
7. Figure 4. Panel A is this from the full eye cup or the retina, RPE, choroid? Or just RPE and choroid?
8. Please show cross sections complementing Figure 5D and 5E.
9. Overall comment regarding multiple genotypes of mice used in the study. The exclusion of mice carrying the crb1 mutation and breeding back to C57BL6J mice is appropriate. However, the use of C57BL6J as a control is a concern, more appropriate is the use of littermate controls. Please clarify if the control mice used in the various experiments were indeed littermate wildtype controls on the C57BL6J background

Referee #2 (Remarks):

The study of Levy et al analysed the mechanism of sub retinal accumulation of monocyte-derived cells and role of ApoE in this process. The topic of the study is relevant and of interest to broad readership. The experimental design is elegant and appropriate animal models are used. The most interesting finding is that ApoE expression in monocyte-derived cells is required for the efficient age, light and laser-induced accumulation of these cells sub retinal space. Next is shown that increased accumulation is due to the decreased apoptosis of subretinal macrophages and their increased survival. The authors provide convincing experimental proofs for the molecular mechanism of this effects that is based on the APOE-mediated induction of IL6 that in turn suppresses expression of FASL. In general, the conclusions are justified by the experimental data, however some additional experiments would strengthen the biological significance of the results

Specific comments

1. It is recommended to examine how wt and ApoE deficient macrophages undergo FAS-induced apoptosis in vitro
2. Additional macrophage markers are commented to use by the analysis of patients material (CD68, CD14, CD163) and perform double IF/confocal microscopy with APOE ab.

Minor comments

1. In the Introduction the authors write that mononuclear phagocytes comprise the family that includes microglial cells, monocytes and macrophages. The microglial cells areonly one of tissue specific resident macrophage subtypes. Other tissue specific macrophages (like Kupffer cells, heart macrophages, fat tissue macrophages, tumor-associated macrophages, and others also belong to the mononuclear phagocytes. The authors have to modified their message accordingly
2. Introduction includes number of details of the state-of-the art. However open questions are not clearly formulated. It is recommended to make a straight forward formulation of gaps of knowledge understandable for the broad readership
3. Introduction, last paragraph. It is strongly recommended to summarize the main message of the study here

© EMBO

Referee #3 (Comments on Novelty/Model System):

In this article, there is an intriguing observation on the association of APOE with MPs. The observations on the mice model of MP accumulation appears to be proving the point that MPs accummulations there contain APOE and therefore there is an inflammatory process associated with some of the pathologies present in AMD eyes. This mice model does not, however, develop AMD like deposits and therefore while the study is interesting I am not sure how this translate to the human work carried out at the beginning of the manuscript especially the association with soft druse. GA is strongly associated with crystalline druse more than soft druse.

One of the surprising omissions of the cited references is the paper by Luhmann at al (DOI: 10.1371/journal.pone.0035551) that elegantly describes the phenotype of the very similar mouse model and the power of using in vivo imaging rather than post mortem imaging presented here. Given tha many poist mortem artefacts this study would have benefited from in vivo imaging and is surprising that this had not been attempted here.

In general the text is overcomplicated, the reader gets lost in abbreviations and acronyms which distracts the message they want to convey. It is commendable that after every very complicated and convoluted results sections there is a clear and unambiguous summary, otherwise even an expert would have been lost.

There are sentences that make no sense: last sentence on page 5; first sentence on 2nd paragraph page 6; POS is photoreceptor outer segment not photoreceptor segment etc...

Clearly and expert team did a great job of conducting a series of experiments that resulted in an important observation, but the text would benefit from simplification and clarity. It is debatable whether there is a need to absolutely force the human AMD issue here and this reviewer is not at all convince that there is need for that.

1st Revision - authors' response 24 November 2014

Referee #1 (Comments on Novelty/Model System):

Identification of potential therapeutic targets of signalling pathways contributing to the late forms of age-related macular degeneration is paramount. The authors outline the involvement of apolipoprotein E, IL-6 and FasL associated pathways in prolonged survival of mononuclear phagocytes in the sub-retinal space, contributing the disease progression. A concern regarding the mice has been raised to authors and essentially involves clarification of control mice used, to include wt mice (littermate controls) rather than C57BL6J mice (from Jax mice) which may not express the same genetic changes that occur with prolonged breeding to a knockout mouse. As such the changes seen may not be due to more than just knockout of the gene of interest (apoE, Cx3cr1, etc).

We agree with the reviewer that genetic background is always a concern in studies using inbred knockout animals. We are aware of this problem, and increasingly so since the discovery of the rd8 mutation in many mouse strains (Chang et al, 2013) compounded with the observation that the mutation's manifestation is highly dependent on modifiers which remain unidentified (Luhmann et al, 2014). We test all of our mouse lines for the most common mutations in C57BL6/J strain by PCR and we regularly backcross individual knockout lines with freshly purchased C57BL6/J mice (Charles River France) to limit the genetic drift that occurs with prolonged breeding. As suggested by the reviewer, we have presented subretinal MP quantification in 12m-old $Cx3cr1^{+/GFP}$, *and* $Cx3cr1^{GFP/GFP}$ littermates of $Cx3cr1^{+/GFP}$ breeders (sFig. 4), demonstrating that the subretinal MP accumulation segregates with the $Cx3cr1^{GFP/GFP}$genotype in littermates. $Cx3cr1^{GFP/GFP}ApoE^{-/-}$-mice were independently generated in two laboratories, that of Dr Combadière, and our own; the animals used in the experiment are subjects of the F4 / F5 generation. The fact that we observed protection in both $Cx3cr1^{GFP/GFP}ApoE^{-/-}$ strains makes it highly likely that *ApoE* deletion is responsible for the protection. This, however, is only "highly likely" as the protective effect might be due to an unknown genetic defect on chromosome 7 close to the APOE gene. The possibility that a phenotype

in a knockout mouse is not due to the knockout but to a spontaneous mutation close to the knocked out gene holds true for any phenotype observed in any knockout animal.

We would also like to point out that our study contains pharmacological experiments (anti-IL-6 antibody, anti-CD14 antibody, and MegaFasL) that corroborate the molecular pathway we deciphered using genetically modified animals.

We added the following paragraph to the results section of "APOE promotes subretinal MP accumulation in Cx3cr1$^{GFP/GFP}$-mice" concerning littermate results and *Cx3cr1*$^{GFP/GFP}$*ApoE*$^{-/-}$strain generation:

"C57BL/6J mice are inbred and carry Pde6b^{rd1} (retinal degeneration 1), Crb1^{rd8} (retinal degeneration 8), Gnat2^{cpfl3} (Cone photoreceptor function loss3) mutations relatively commonly (Chang et al, 2013). These mutations can lead to subretinal inflammation secondary to primary retinal degeneration (Luhmann et al, 2012). In our experiments all mice strains used tested negative for these three mutations. Furthermore, subretinal MP accumulation in 12m-old *Cx3cr1*$^{+/GFP}$*, and Cx3cr1*$^{GFP/GFP}$ littermates of *Cx3cr1*$^{+/GFP}$ breeders (sFig. 4) showed no evidence of the influence of an unknown contributor gene specific to the *Cx3cr1*$^{GFP/GFP}$mouse line (sFig. 4). *Cx3cr1*$^{GFP/GFP}$*ApoE*$^{-/-}$-mice were generated twice with independently purchased *Cx3cr1*$^{GFP/GFP}$ and *ApoE*$^{-/-}$-mice (once at the *Laboratoire Immunité et Infection* and once at the *Institut de la Vision*) and both *Cx3cr1*$^{GFP/GFP}$*ApoE*$^{-/-}$-mice strains were protected against the subretinal MP accumulation observed in the two *Cx3cr1*$^{GFP/GFP}$mouse strains of the two sites. Taken together, these results make it highly unlikely that the MP accumulation in *Cx3cr1*$^{GFP/GFP}$mice and the protection in *Cx3cr1*$^{GFP/GFP}$*ApoE*$^{-/-}$-mice are due to genes other than *Cx3cr1* and *ApoE*."

Referee #1 (Remarks):

Overall comments:

In this manuscript Levy et al. report on features of subretinal mononuclear phagocytes (MPs), which are reported to infiltrate the subretinal space in AMD patients (by this group and others). They find that subretinal MPs in AMD patients express apoE. In Cx3Cr1-/- mice they confirm their previous finding of increased subretinal MPs, extending it with the observation that these MPs also express high levels of apoE in this genetic model, and that apoE deletion reverses this pathology. Mechanistically they suggest that the infiltration and prolonged survival of these MPs occurs through activation of CD14 and increased expression of IL-6 due to increased apoE expression. Given that the identification of potential therapeutic targets of signalling pathways contributing to the late forms of age-related macular degeneration (especially GA) is an important unmet need, the reported findings of the involvement of apoE, IL6 and FasL associated pathways in prolonged survival of MPs in the subretinal space, are significant additions to the field of AMD research and of high importance.

We thank the reviewer for such supportive comments.

Specific comments:

1. Introduction-1st paragraph: drusen accumulate between the RPE basal lamina and the inner collagenous layer of Bruch's. As such they are within Bruch's membrane, not "located on the Bruch's membrane". Please correct.

We corrected this mistake in the introduction:

"…which are located <u>in</u> the Bruch's membrane (BM) and partially covered by the retinal pigment epithelium (RPE) (Sarks, 1976)…"

2. Figure 1. The flatmount images provide a very nice overview of the distribution of IBA1+ cells. However, cross-sections would illustrate co-localization of apoE protein within subretinal IBA1+ cells better and is recommended, given this study hinges on the premise that subretinal MPs "strongly express apoE".

As suggested, the new figure 2, entirely dedicated to APOE expression in macrophages and in AMD, presents immunohistochemistry on both flatmounts and cross-sections. The new figure additionally shows a) a positive control (APOE/IBA1 double labelling of sections on human tonsil samples, extracted for recurrent acute tonsillitis) b) APOE and APOE/IBA1 double labelling on paraffin sections of human donor tissues with GA.

For the human GA studies on sections (which inevitably contain RPE), we visualized APOE using an enzyme/substrate revelation system (FAST RED). In human tissue from elderly subjects the RPE is highly autofluorescent. The FAST RED revelation system was used to avoid any possible confusion between APOE expression within the RPE and RPE autofluorescence. In GA sections, we also marked subretinal macrophages using an APOE/IBA1 double labelling procedure. Subretinal macrophages are strongly IBA1 positive and can be visualized using a fluorescent secondary antibody. The RPE autofluorescence was captured using a filter that visualizes far-red emission.

These new results are presented in the new figure 2 and described in the dedicated results section.

3. Panels E-H within the last two paragraphs of the "subretinal MPs accumulate in AMD and express apoE" results section are incorrectly referred to, please correct.

This is corrected in the new version of the manuscript.

4. Figure 2G. Please provide a control reference for the western blot. Another secreted protein?

Western blot analysis of soluble Mer receptor tyrosine kinase that is released constitutively from cultured macrophages (Sather et al, 2007) is presented as a loading control in the revised manuscript.

5. Provide detailed description of quantification methods used for Figure 2, panels L-N. How far out of the lesion were MPs counted? Etc...

We apologize for this unintended omission; it was indeed not specified how the subretinal MPs surrounding laser impacts were quantified. We counted IBA1+cells on the RPE at a distance of 0-500µm from the CD102+ CNVs. This is now mentioned in the corresponding result section and figure.

6. Figure 3. What is the quantification measure in the charts? Number of cells/eye? %+/eye? All normalized to C57BL/6J? Please clarify.

We counted $CFSE^{+}F4/80^{+}$cells /eye. This was missing in the former figures and has now been corrected in all the figures that present data from macrophage adoptive transfer experiments.

7. Figure 4. Panel A is this from the full eye cup or the retina, RPE, choroid? Or just RPE and choroid?

We thank the reviewer for this remark. The present RT-PCRs were performed on RPE/choroidal extracts. This is now clearly stated in the results section and the figure legend.

8. Please show cross sections complementing Figure 5D and 5E.

We agree that the IL6/IBA1 double labelling on RPE/choroidal flatmounts failed to show the tissue context, as the RPE was not visualized. To correct this, we have presented IL6/IBA1 double labelling in which we also visualized the RPE using phalloidin. Orthogonal z-stack projections of confocal microscopy are shown to illustrate the close proximity of IL6 expressing subretinal $IBA1^+$MPs with the RPE.

9. Overall comment regarding multiple genotypes of mice used in the study. The exclusion of mice carrying the crb1 mutation and breeding back to C57BL6J mice is appropriate. However, the use of C57BL6J as a control is a concern, more appropriate is the use of littermate controls. Please clarify if the control mice used in the various experiments were indeed littermate wildtype controls on the C57BL6J background

As mentioned above in greater detail, we share the reviewer's concern about appropriate controls in knockout experiments. We present littermate data for the subretinal MP accumulation data in *Cx3cr1*$^{GFP/GFP}$mice in the new supplementary Figure 4. As mentioned above, in the new manuscript we have added a paragraph in the "APOE promotes subretinal MP accumulation in Cx3cr1$^{GFP/GFP}$-mice" section discussing possible strain-related artifacts.

Referee #2 (Remarks):

The study of Levy et al analysed the mechanism of sub retinal accumulation of monocyte-derived cells and role of ApoE in this process. The topic of the study is relevant and of interest to broad readership. The experimental design is elegant and appropriate animal models are used. The most interesting finding is that ApoE expression in monocyte-derived cells is required for the efficient age, light and laser-induced accumulation of these cells sub retinal space. Next is shown that increased accumulation is due to the decreased apoptosis of subretinal macrophages and their increased survival. The authors provide convincing experimental proofs for the molecular mechanism of this effects that is based on the APOE-mediated induction of IL6 that in turn suppresses expression of FASL. In general, the conclusions are justified by the experimental data, however some additional experiments would strengthen the biological significance of the results

We thank the reviewer for their kind remarks.

Specific comments

1. It is recommended to examine how wt and ApoE deficient macrophages undergo FAS-induced apoptosis in vitro

Indeed, in the first version of the manuscript, we only analyzed the in vitro susceptibility to FAS-activation induced apoptosis in wildtype and Cx3cr1. In response to the reviewer's comment we exposed monocytes and thiglycollate-elicited peritoneal macrophages of wildtype-, *Cx3cr1*$^{GFP/GFP}$-, *Cx3cr1*$^{GFP/GFP}$*ApoE*$^{-/-}$- *and ApoE*$^{-/-}$-cells to MegaFasL (1ng /ml) and quantified $TUNEL^+$cells at 24h *in vitro* (Fig. 5K). Our results confirm previous reports that Mos are more susceptible to FasL induced apoptosis *in vitro* compared to Mfs that are rather resistant (Kiener et al, 1997; Park et al, 2003; Um et al, 1996). We did not observe a difference between wildtype- and *Cx3cr1*$^{GFP/GFP}$-cells of either Mos or Mfs, nor did we observe dramatic differences between genotypes all together. However, we noted a tendency of increased susceptibility in Mos of both *Cx3cr1*$^{GFP/GFP}$*ApoE*$^{-/-}$- *and ApoE*$^{-/-}$-cells, which might participate in the differences in clearance observed *in vivo*. On the other hand, *Cx3cr1*$^{GFP/GFP}$*ApoE*$^{-/-}$- *and ApoE*$^{-/-}$- Mfs were slightly less sensitive to MegaFasL-induced apoptosis in vitro and apoptosis could not be reproducibly induced in these cells in vitro. These

results highlight that FasL acts in concert with other factors to induce Mf apoptosis in the subretinal space, as the effect of MegaFasL on subretinally clearance of Mfs was much stronger than MegaFasL–induced apoptosis *in vitro*. Indeed, this synergistic effect of FasL with other RPE-derived factors in the induction of leukocyte apoptosis was already suggested by the following observations: FASL competent, but not FASL-deficient, RPE allografts to non-immune privileged sites suppress their elimination by effector-cells (T-cells and macrophages) and survive prolonged periods of time (Wenkel & Streilein, 2000). However FASL over-expression alone in an allograft, such as a Langerhans cell graft, is not sufficient to induce leukocyte apoptosis and convey immune privilege (Kang et al, 1997). Therefore, RPE FASL expression in RPE seems a necessary, but not sufficient factor to induce leukocyte apoptosis; other factors expressed by the RPE must be involved.

The in vitro results are presented in the figure and results section « Fas-FasL signalling mediates subretinal MP clearance"

2. Additional macrophage markers are commented to use by the analysis of patients material (CD68, CD14, CD163) and perform double IF/confocal microscopy with APOE ab.

We included an IBA1/CD18 double labelling on human retinal flatmounts to confirm the MP nature of subretinal IBA1+ cells in the new figure 1. We chose CD18 as it is equally expressed in monocytes and microglial cells, the two populations that make up the majority of subretinal MPs (Sennlaub et al, 2013). Furthermore, the new figure 2 includes IBA1/APOE double labelling of tonsil samples (positive control) and of sections from GA patients additionally to the immunohistochemistry on retinal flat mounts, as described above (point 2 of reviewer 1).

Minor comments

1. In the Introduction the authors write that mononuclear phagocytes comprise the family that includes microglial cells, monocytes and macrophages. The microglial cells areonly one of tissue specific resident macrophage subtypes. Other tissue specific macrophages (like Kupffer cells, heart macrophages, fat tissue macrophages, tumor-associated macrophages, and others also belong to the mononuclear phagocytes. The authors have to modified their message accordingly

We apologize for this confusion. We thought that "includes" signified our list was not exhaustive. We hope that stating : "Mononuclear phagocytes (MP) comprise a family of cells that include microglial cells (MC), monocytes (Mo), and macrophages (Mϕ), among others" will make this clearer.

2. Introduction includes number of details of the state-of-the art. However open questions are not clearly formulated. It is recommended to make a straight forward formulation of gaps of knowledge understandable for the broad readership

3. Introduction, last paragraph. It is strongly recommended to summarize the main message of the study here

The revised introduction now more clearly states open questions, such as "The reasons for the breakdown of subretinal immunosuppression and accumulation of MPs in AMD remain unknown", or "It is currently not clear how APOE and IL-6 participate in AMD pathogenesis." The introduction now ends a paragraph that resumes the findings of the paper, as suggested by the reviewer.

Referee #3 (Comments on Novelty/Model System):

In this article, there is an intriguing observation on the association of APOE with MPs. The observations on the mice model of MP accumulation appears to be proving the point that MPs accummulations there contain APOE and therefore there is an inflammatory process associated

with some of the pathologies present in AMD eyes. This mice model does not, however, develop AMD like deposits and therefore while the study is interesting I am not sure how this translate to the human work carried out at the beginning of the manuscript especially the association with soft druse. GA is strongly associated with crystalline druse more than soft druse.

We thank the reviewer for his positive remarks. AMD is a multifactorial disease and no single mechanism has been identified as sufficient cause for the disease. Risk factors such as age, smoking, and a family history of AMD have been shown to predispose subjects to AMD. Given the multifactorial nature of the disease and the fact that none of the risk factors, taken alone, are sufficient to trigger the disease, it is to be expected that no animal model mimicking a single risk factor or combination of two risk factors (such as age plus another factor) are sufficient to reproduce all aspects of the disease. However, such models show strong potential for modeling different aspects of AMD and provide a point of entry toward understanding AMD's pathomechanism(s). The human data presented here illustrates that MP do not accumulate in contact with healthy RPE but do accumulate in contact with the RPE adjacent to GA lesions and large drusen. *Cx3cr1*$^{GFP/GFP}$-mice do not develop drusen and RPE atrophy, as the reviewer rightly points out, but they do model the MP accumulation on the RPE that is observed around drusen and atrophic zones (Combadiere et al, 2007; Sennlaub et al, 2013) Interestingly, human subretinal MPs and subretinal MPs observed in *Cx3cr1*$^{GFP/GFP}$-mice both strongly express APOE. The *Cx3cr1*$^{GFP/GFP}$-mouse model therefore promises insight concerning the effect of APOE expressed by subretinal MPs, as developed in the paper.

Concerning soft drusen, we apologize for having used the term "soft" drusen, which is a clinical description of the fundus appearance of the large drusen. On the RPE flatmounts used for the immunohistochemistry, we can accurately state the drusen are large (>125mm) but not whether they appeared in the fundus as "soft". This has been corrected in the new manuscript. Large drusen are an important risk factor for the development of late AMD (Klein et al, 2004).

One of the surprising omissions of the cited references is the paper by Luhmann at al (DOI: 10.1371/journal.pone.0035551) that elegantly describes the phenotype of the very similar mouse model and the power of using in vivo imaging rather than post mortem imaging presented here. Given tha many poist mortem artefacts this study would have benefited from in vivo imaging and is surprising that this had not been attempted here.

We agree with the reviewer that the cited article elegantly describes the in vivo quantification of autofluorescence dots and white dots in fundus photography that are caused by ingestion of POS by subretinal MPs, a phenomenon we have also observed in *Cx3cr1*$^{GFP/GFP}$-mice (Combadiere et al, 2007; Sennlaub et al, 2013). The Luhmann paper also very convincingly shows the possible effect of the rd8 mutation on subretinal inflammation and the new manuscript cites it in this context ("… mutations can lead to subretinal inflammation secondary to primary retinal degeneration (Luhmann et al, 2012)").

However, while all autofluorescent/white dots observed in vivo are caused by subretinal MPs that have ingested POS, subretinal MPs that have not ingested POS remain invisible in in vivo imaging. The quantification of autofluorescent/white dots by SLO and fundus pictures therefore only corresponds to a subpopulation of subretinal MPs. Furthermore, in vivo imagery allows only for accurate visualization of the central retina and is less useful for observing the peripheral retina. These considerations directed our choice to quantify the numbers of subretinal MPs on post-mortem IBA-1 stained retinal and RPE/choroidal flatmounts rather than in vivo quantification techniques. Over the years we have performed hundreds of flatmount stainings/quantifications; we are very confident that the subretinal MPs we identify with the IBA-1 stain are not polluted by post-mortem artifacts.

In general the text is overcomplicated, the reader gets lost in abbreviations and acronyms which distracts the message they want to convey. It is commendable that after every very complicated and convoluted results sections there is a clear and unambiguous summary, otherwise even an expert would have been lost. There are sentences that make no sense: last sentence on page 5; first sentence on 2nd paragraph page 6; POS is photoreceptor outer segment not photoreceptor segment etc...

Clearly and expert team did a great job of conducting a series of experiments that resulted in an important observation, but the text would benefit from simplification and clarity. It is debatable whether there is a need to absolutely force the human AMD issue here and this reviewer is not at all convince that there is need for that.

We would like to thank the reviewer for his positive evaluation. We simplified, corrected and streamlined the manuscript. In particular we have simplified the description of the adoptive transfer experiments and have cut the use of abbreviations to a strict minimum.

References:

Chang B, Hurd R, Wang J, Nishina P (2013) Survey of common eye diseases in laboratory mouse strains. *Invest Ophthalmol Vis Sci* **54**(7)**:** 4974-4981

Combadiere C, Feumi C, Raoul W, Keller N, Rodero M, Pezard A, Lavalette S, Houssier M, Jonet L, Picard E, Debre P, Sirinyan M, Deterre P, Ferroukhi T, Cohen SY, Chauvaud D, Jeanny JC, Chemtob S, Behar-Cohen F, Sennlaub F (2007) CX3CR1-dependent subretinal microglia cell accumulation is associated with cardinal features of age-related macular degeneration. *J Clin Invest* **117**(10)**:** 2920-2928

Kang SM, Schneider DB, Lin Z, Hanahan D, Dichek DA, Stock PG, Baekkeskov S (1997) Fas ligand expression in islets of Langerhans does not confer immune privilege and instead targets them for rapid destruction. *Nat Med* **3**(7)**:** 738-743

Kiener PA, Davis PM, Starling GC, Mehlin C, Klebanoff SJ, Ledbetter JA, Liles WC (1997) Differential induction of apoptosis by Fas-Fas ligand interactions in human monocytes and macrophages. *J Exp Med* **185**(8)**:** 1511-1516

Klein R, Peto T, Bird A, Vannewkirk MR (2004) The epidemiology of age-related macular degeneration. *Am J Ophthalmol* **137**(3)**:** 486-495

Luhmann UF, Carvalho LS, Holthaus SM, Cowing JA, Greenaway S, Chu CJ, Herrmann P, Smith AJ, Munro PM, Potter P, Bainbridge JW, Ali RR (2014) The severity of retinal pathology in homozygous Crb1rd8/rd8 mice is dependent on additional genetic factors. *Hum Mol Genet*

Luhmann UF, Lange CA, Robbie S, Munro PM, Cowing JA, Armer HE, Luong V, Carvalho LS, MacLaren RE, Fitzke FW, Bainbridge JW, Ali RR (2012) Differential modulation of retinal degeneration by Ccl2 and Cx3cr1 chemokine signalling. *PLoS One* **7**(4)**:** e35551

Park DR, Thomsen AR, Frevert CW, Pham U, Skerrett SJ, Kiener PA, Liles WC (2003) Fas (CD95) induces proinflammatory cytokine responses by human monocytes and monocyte-derived macrophages. *J Immunol* **170**(12)**:** 6209-6216

Sarks SH (1976) Ageing and degeneration in the macular region: a clinico-pathological study. *Br J Ophthalmol* **60**(5)**:** 324-341

Sather S, Kenyon KD, Lefkowitz JB, Liang X, Varnum BC, Henson PM, Graham DK (2007) A soluble form of the Mer receptor tyrosine kinase inhibits macrophage clearance of apoptotic cells and platelet aggregation. *Blood* **109**(3)**:** 1026-1033

Sennlaub F, Auvynet C, Calippe B, Lavalette S, Poupel L, Hu SJ, Dominguez E, Camelo S, Levy O, Guyon E, Saederup N, Charo IF, Rooijen NV, Nandrot E, Bourges JL, Behar-Cohen F, Sahel JA, Guillonneau X, Raoul W, Combadiere C (2013) CCR2(+) monocytes infiltrate atrophic lesions in age-related macular disease and mediate photoreceptor degeneration in experimental subretinal inflammation in Cx3cr1 deficient mice. *EMBO Mol Med* **5**(11)**:** 1775-1793

Um HD, Orenstein JM, Wahl SM (1996) Fas mediates apoptosis in human monocytes by a reactive oxygen intermediate dependent pathway. *J Immunol* **156**(9)**:** 3469-3477

Wenkel H, Streilein JW (2000) Evidence that retinal pigment epithelium functions as an immune-privileged tissue. *Invest Ophthalmol Vis Sci* **41**(11)**:** 3467-3473

2nd Editorial Decision 09 December 2014

Thank you for the submission of your revised manuscript to EMBO Molecular Medicine. We have now received the enclosed reports from the Reviewers that were asked to re-assess it. As you will see the Reviewers are now globally supportive although there are a few remaining issues.

Briefly, both Reviewer 1 and 2 mention a few issues that require clarification and relative amendments in the manuscript. I am prepared to make an Editorial decision on your final, revised version, provided the issues raised are dealt with as requested. When you do submit, please upload an additional copy of your manuscript with the changes clearly marked.

In the likely event of acceptance, you will be asked to fulfill a number of editorial requirements as listed below. I suggest that you provide the following information and amendments requested directly with the next, final version of your manuscript:

1) Please provide "The Paper Explained" section, which is still missing. As you know, EMBO Molecular Medicine articles are accompanied by a structured summary of the article to emphasize the major findings of the paper and their medical implications for the non-specialist reader. Please provide a summary accessible to non-specialists and specialists alike, highlighting the medical issue you are addressing (heading: PROBLEM), the results obtained (heading: RESULTS), and their clinical impact (heading: IMPACT). This may be edited to ensure that readers understand the significance and context of the research. You may refer to any of our published articles as a reference (embomolmed.org).

2) For experiments involving human subjects the authors must identify the committee approving the experiments and include a statement that informed consent was obtained from all subjects and that the experiments conformed to the principles set out in the WMA Declaration of Helsinki [http://www.wma.net/en/30publications/10policies/b3/] and the NIH Belmont Report [http://ohsr.od.nih.gov/guidelines/belmont.html]. Any restrictions on the availability or on the use of human data or samples should be clearly specified in the manuscript. Any restrictions that may detract from the overall impact of a study or undermine its reproducibility will be taken into account in the editorial decision.

3) We are now encouraging the publication of source data, particularly for electrophoretic gels and blots, with the aim of making primary data more accessible and transparent to the reader. Would you be willing to provide a PDF file per figure that contains the original, uncropped and unprocessed scans of all or at least the key gels used in the manuscript? The PDF files should be labeled with the appropriate figure/panel number, and should have molecular weight markers; further annotation may be useful but is not essential. The PDF files will be published online with the article as supplementary "Source Data" files. If you have any questions regarding this just contact me.

4) Every published paper now includes a 'Synopsis' to further enhance discoverability. Synopses are displayed on the journal webpage and are freely accessible to all readers. They include a short standfirst (to be written by the Editor) as well as 2-5 one sentence bullet points that summarise the paper (to be written by the author). Therefore, please provide the short list of bullet points that summarise the key NEW findings. The bullet points should be designed to be complementary to the abstract - i.e. not repeat the same text. We encourage inclusion of key acronyms and quantitative information. Please use the passive voice. Please attach these in a separate file or send them by email, we will incorporate them accordingly.

I look forward to seeing a revised form of your manuscript soon.

***** Reviewer's comments *****

Referee #1 (Remarks):

Most of my previous comments and concerns have been addressed quite nicely by the authors. Below are additional minor questions and comments:

1. There are statements in the third paragraph on the third page of the introduction that are not entirely accurate and should be corrected. The APOE4 transgenic mice reported to "develop lipid accumulation in BM, which are proposed as being similar to early AMD" do indeed have slightly less apoE though it is does not appear to be statistically significant, as shown in a supplementary figure of that paper. However, the "AMD' pathology is seen only in mice also fed a high fat diet and the text as written has omitted this important point. Similarly the apoE knock mouse study explores the additional insult of feeding the mice a cholesterol rich diet. Please correct text accordingly. Importantly, are there any studies that have compared circulating levels of apoE and AMD progression? Important to note that in the Introduction as well.

2. Figure 1: On page 1 of results, the authors state they observed numerous "IBA1+ cells within drusen", yet in the last sentence of page 1 continuing onto page 2 of the results, they state "....subretinal MPs in AMD and illustrate their accumulation around large drusen". Which is correct? If the former then the finding of numerous IBA1+ cells "in" drusen is quite novel and once again a cross sectional demonstration of IBA1+ cell immunolocalized within drusen is needed to add support. I believe, MP processes extending into drusen have been observed before (with EM), but they have been quite hard to show and certainly not 'numerous'.

3. Page 5 of results: Do C57BL/6J mice carry the RD8 mutation? I thought the 6Js didn't but the 6Ns and other mice did? Please correct the first sentence of the second paragraph on this page.

4. Last sentence of the last paragraph needs a minor addition of the word "in". Taken together, the results of our study.... that may participate "in" the weakend...

Referee #2 (Comments on Novelty/Model System):

The revised version is significantly improved. However, the experimental data provided to answer Point 1 of my original review indicate broader range of the mechanisms that are responsible for the observed effects in vivo
Therefore, the explanation provided in the answers to my comment 1 should be adequately integrated in the Discussion

Referee #3 (Comments on Novelty/Model System):

This is a very interesting and very detailed study with a potentially important outcome. How suitable this will be for medical intervention will need to come from further research hence the medium for medical impact.

Referee #3 (Remarks):

I am satisfied that the Authors addressed the comments I made earlier and and found no further issues with the manuscript. I do need to add that I am out of my dept when it comes to technical details.

2nd Revision - authors' response 11 December 2014

Point-by-point response to the reviewers' comments

Referee #1:

Most of my previous comments and concerns have been addressed quite nicely by the authors. Below are additional minor questions and comments:

1. There are statements in the third paragraph on the third page of the introduction that are not entirely accurate and should be corrected. The APOE4 transgenic mice reported to "develop lipid accumulation in BM, which are proposed as being similar to early AMD" do indeed have slightly less apoE though it is does not appear to be statistically significant, as shown in a supplementary figure of that paper. However, the "AMD' pathology is seen only in mice also fed a high fat diet and the text as written has omitted this important point. Similarly the apoE knock mouse study explores the additional insult of feeding the mice a cholesterol rich diet. Please correct text accordingly. Importantly, are there any studies that have compared circulating levels of apoE and AMD progression? Important to note that in the Introduction as well.

We thank the reviewer for these insightful remarks and we corrected the paragraph in the introduction accordingly:

"On the other hand, *ApoE*$^{-/-}$ mice that were fed a high fat diet develop lipid accumulations in BM, which are proposed as being similar to early AMD (Ong et al, 2001). *APOE4* transgenic mice fed a high fat diet develop similar deposits (Malek et al, 2005) even though the APOE concentration observed in *APOE4* transgenic mice are only marginally decreased in their plasma (Riddell et al, 2008) and similar in the CSF (Riddell et al, 2008) and retina (Malek et al, 2005) compared to *APOE3* mice. The structural change in the APOE4 protein however leads to diminished association for HDL (Dong & Weisgraber, 1996) and impaired reverse cholesterol transport (Heeren et al, 2004). Low APOE concentrations or impaired reverse cholesterol transport could thereby hinder efficient lipid evacuation from the RPE to the choroid and lead to drusen development. This hypothesis is however in contradiction with the APOE accumulation observed in AMD donor eyes (Anderson et al, 2001; Klaver et al, 1998) and the protective effect of the *APOE4* allele in AMD (McKay et al, 2011)."

2. Figure 1: On page 1 of results, the authors state they observed numerous "IBA1+ cells within drusen", yet in the last sentence of page 1 continuing onto page 2 of the results, they state "....subretinal MPs in AMD and illustrate their accumulation around large drusen". Which is correct? If the former then the finding of numerous IBA1+ cells "in" drusen is quite novel and once again a cross sectional demonstration of IBA1+ cell immunolocalized within drusen is needed to add support. I believe, MP processes extending into drusen have been observed before (with EM),

but they have been quite hard to show and certainly not 'numerous'.

We thank the reviewer for raising this important point. We do indeed observe IBA-1$^+$cells within large drusen, confirming the presence of MPs within large drusen that we previously published using CCR2 immunohistochemistry on paraffin sections (Sennlaub et al, 2013). Hageman et al. previously published two micrographs of HLA-DR and CD68 immunohistochemistry depicting MP cellular parts in drusen (Fig. 10 b and c (Hageman et al, 2001)). The panels lack a scale bar, but judging from the visible RPE (its thickness, ≈10µm, can serve as a measure of size), the shown drusen are small and dome-shaped. A detailed analysis of MPs within drusen of different sizes is ongoing in our laboratory, but is beyond the scope of this study. W

e reformulated the relevant parts in the result section:

"Furthermore, IBA-1$^+$cells were detected on the RPE adjacent to large drusen (>125mm), visible under the dissecting microscope as pale lesions after removal of the retina (Fig. 1C, inset) and as dome-shaped protrusions under the confocal microscope (Fig. 1C, oblique projection of a Z-stack; Fig. 1D, orthogonal Z-stack projection). Double-labeling on the subretinal side of the overlaying retina (to avoid masking by RPE autofluorescence) shows that subretinal IBA-1$^+$MPs (Fig. 1E green fluorescence) also express the pan-MP marker CD18 (Fig. 1F red fluorescence, Fig. 1G merge). IBA-1$^+$MPs in close contact with the RPE (Fig. 1H, lateral Z-stack projections) were observed in the vicinity of all examined large drusen and atrophic zones. Interestingly, we also detected IBA-1$^+$MPs within the large drusen, confirming our previous immunohistochemical detection of CCR2$^+$MPs on paraffin sections within large drusen (Sennlaub et al, 2013). HLA-DR and CD68 positive MP dendrites have previously been observed in smaller, dome-shaped drusen (Hageman et al, 2001). A detailed analysis of MPs within drusen of different sizes is ongoing in our laboratory, but beyond the scope of this study.

These observations considered together confirm the presence of subretinal MPs in AMD (Gupta et al, 2003; Penfold et al, 1985; Sennlaub et al, 2013) and illustrate their presence in contact with the RPE around large drusen and GA lesions. They are very rare in healthy donors. This further suggests that RPE-mediated immunosuppression is impaired in intermediate AMD (large drusen) and late AMD (GA)."

3. Page 5 of results: Do C57BL/6J mice carry the RD8 mutation? I thought the 6Js didn't but the 6Ns and other mice did? Please correct the first sentence of the second paragraph on this page.

We are sorry for this mistake. C57BL/6 mice relatively commonly carry these mutations. They are indeed very common in the C57BL/6N substrain and less so in the C57BL/6J strain. But we have come across homozygote *Crb1*^{rd8} carriers in transgenic mice on a "pure" C57BL/6J background (TSP-1$^{-/-}$mice from the Jackson laboratory)! We have reformulated the new version of the manuscript:

"C57BL/6 mice are inbred and can carry *Pde6b*^{rd1} (retinal degeneration 1), *Crb1*^{rd8} (retinal degeneration 8), *Gnat2*^{cpfl3} (Cone photoreceptor function loss3) mutations relatively commonly (Chang et al, 2013)."

4. Last sentence of the last paragraph needs a minor addition of the word "in". Taken together, the results of our study.... that may participate "in" the weakend...

We are sorry for this mistake. We have corrected the new version of the manuscript:

Referee #2 (Remarks):

The revised version is significantly improved. However, the experimental data provided to answer Point 1 of my original review indicate broader range of the mechanisms that are responsible for the observed effects in vivo

Therefore, the explanation provided in the answers to my comment 1 should be adequately integrated in the Discussion

We thank the reviewer for his kind remarks. Additionally to the description and discussion of the results of FASL-induced apoptosis of monocyte and macrophage in the results section of Figure 5, we added a sentence in the discussion:

"Interestingly, *ApoE*-deficient monocytes, but not macrophages, also showed a tendency toward increased susceptibility to FASL-induced apoptosis *in vitro*, which might contribute toward the differences in clearance observed *in vivo."*

References:

Anderson DH, Ozaki S, Nealon M, Neitz J, Mullins RF, Hageman GS, Johnson LV (2001) Local cellular sources of apolipoprotein E in the human retina and retinal pigmented epithelium: implications for the process of drusen formation. *Am J Ophthalmol* **131:** 767-781

Chang B, Hurd R, Wang J, Nishina P (2013) Survey of common eye diseases in laboratory mouse strains. *Invest Ophthalmol Vis Sci* **54:** 4974-4981

Dong LM, Weisgraber KH (1996) Human apolipoprotein E4 domain interaction. Arginine 61 and glutamic acid 255 interact to direct the preference for very low density lipoproteins. *J Biol Chem* **271:** 19053-19057

Gupta N, Brown KE, Milam AH (2003) Activated microglia in human retinitis pigmentosa, late-onset retinal degeneration, and age-related macular degeneration. *Exp Eye Res* **76:** 463-471

Hageman GS, Luthert PJ, Victor Chong NH, Johnson LV, Anderson DH, Mullins RF (2001) An integrated hypothesis that considers drusen as biomarkers of immune-mediated processes at the RPE-Bruch's membrane interface in aging and age-related macular degeneration. *Prog Retin Eye Res* **20:** 705-732

Heeren J, Grewal T, Laatsch A, Becker N, Rinninger F, Rye KA, Beisiegel U (2004) Impaired recycling of apolipoprotein E4 is associated with intracellular cholesterol accumulation. *J Biol Chem* **279:** 55483-55492

Klaver CC, Kliffen M, van Duijn CM, Hofman A, Cruts M, Grobbee DE, van Broeckhoven C, de Jong PT (1998) Genetic association of apolipoprotein E with age-related macular degeneration. *Am J Hum Genet* **63:** 200-206

Malek G, Johnson LV, Mace BE, Saloupis P, Schmechel DE, Rickman DW, Toth CA, Sullivan PM, Bowes Rickman C (2005) Apolipoprotein E allele-dependent pathogenesis: a model for age-related retinal degeneration. *Proc Natl Acad Sci U S A* **102:** 11900-11905

McKay GJ, Patterson CC, Chakravarthy U, Dasari S, Klaver CC, Vingerling JR, Ho L, de Jong PT, Fletcher AE, Young IS, Seland JH, Rahu M, Soubrane G, Tomazzoli L, Topouzis F, Vioque J, Hingorani AD, Sofat R, Dean M, Sawitzke J, Seddon JM, Peter I, Webster AR, Moore AT, Yates JR, Cipriani V, Fritsche LG, Weber BH, Keilhauer CN, Lotery AJ, Ennis S, Klein ML, Francis PJ, Stambolian D, Orlin A, Gorin MB, Weeks DE, Kuo CL, Swaroop A, Othman M, Kanda A, Chen W, Abecasis GR, Wright AF, Hayward C, Baird PN, Guymer RH, Attia J, Thakkinstian A, Silvestri G

(2011) Evidence of association of APOE with age-related macular degeneration: a pooled analysis of 15 studies. *Hum Mutat* **32:** 1407-1416

Ong JM, Zorapapel NC, Rich KA, Wagstaff RE, Lambert RW, Rosenberg SE, Moghaddas F, Pirouzmanesh A, Aoki AM, Kenney MC (2001) Effects of cholesterol and apolipoprotein E on retinal abnormalities in ApoE-deficient mice. *Invest Ophthalmol Vis Sci* **42:** 1891-1900

Penfold PL, Killingsworth MC, Sarks SH (1985) Senile macular degeneration: the involvement of immunocompetent cells. *Graefes Arch Clin Exp Ophthalmol* **223:** 69-76

Riddell DR, Zhou H, Atchison K, Warwick HK, Atkinson PJ, Jefferson J, Xu L, Aschmies S, Kirksey Y, Hu Y, Wagner E, Parratt A, Xu J, Li Z, Zaleska MM, Jacobsen JS, Pangalos MN, Reinhart PH (2008) Impact of apolipoprotein E (ApoE) polymorphism on brain ApoE levels. *J Neurosci* **28:** 11445-11453

Sennlaub F, Auvynet C, Calippe B, Lavalette S, Poupel L, Hu SJ, Dominguez E, Camelo S, Levy O, Guyon E, Saederup N, Charo IF, Rooijen NV, Nandrot E, Bourges JL, Behar-Cohen F, Sahel JA, Guillonneau X, Raoul W, Combadiere C (2013) CCR2(+) monocytes infiltrate atrophic lesions in age-related macular disease and mediate photoreceptor degeneration in experimental subretinal inflammation in Cx3cr1 deficient mice. *EMBO Mol Med* **5:** 1775-1793
